# G-quadruplexes as a source of vulnerability in BRCA2*-*deficient granule cell progenitors and medulloblastoma

**DOI:** 10.1073/pnas.2503872122

**Published:** 2025-08-25

**Authors:** Danielle L. Keahi, Mathijs A. Sanders, Matthew R. Paul, Andrew L. H. Webster, Yin Fang, Tom F. Wiley, Samer Shalaby, Thomas S. Carroll, Settara C. Chandrasekharappa, Carolina Sandoval-Garcia, Margaret L. MacMillan, John E. Wagner, Mary E. Hatten, Agata Smogorzewska

**Affiliations:** ^a^Laboratory of Genome Maintenance, The Rockefeller University, New York, NY 10065; ^b^Cancer, Ageing and Somatic Mutation, Wellcome Sanger Institute, Hinxton CB10 1SA, United Kingdom; ^c^Department of Hematology, Erasmus MC Cancer Institute, Rotterdam 3015 GD, The Netherlands; ^d^Bioinformatics Resource Center, The Rockefeller University, New York, NY 10065; ^e^Laboratory of Developmental Neurobiology, The Rockefeller University, New York, NY 10065; ^f^Comparative Bioscience Center, The Rockefeller University, New York, NY 10065; ^g^Flow Cytometry Resource Center, The Rockefeller University, New York, NY 10065; ^h^Cancer Genetics and Comparative Genomics Branch, National Human Genome Research Institute, National Institutes of Health, Bethesda, MD 20892; ^i^Department of Neurosurgery, University of Minnesota, Minneapolis, MN 55455; ^j^Department of Pediatrics, University of Minnesota, Minneapolis, MN 55455

**Keywords:** BRCA2, medulloblastoma, PIF1, G-quadruplexes, granule cell progenitors

## Abstract

Biallelic pathogenic variants in the essential DNA repair gene *BRCA2* cause Fanconi anemia complementation group D1. Patients in this group are highly prone to develop embryonal tumors, most commonly medulloblastoma arising from the cerebellar granule cell progenitors (GCPs). GCPs undergo high proliferation in the postnatal cerebellum under Sonic Hedgehog (SHH) activation, but the type of DNA lesions that require the function of the BRCA2 to prevent tumorigenesis remains unknown. To identify such lesions, we assessed both GCP neurodevelopment and tumor formation using a mouse model with deletion of exons three and four of *Brca2* in the central nervous system, coupled with global *Trp53* loss. *Brca2^Δex3-4^;Trp53^−/−^* animals developed SHH subgroup medulloblastomas with complete penetrance. Whole-genome sequencing of the tumors identified structural variants with breakpoints enriched in areas overlapping putative G-quadruplexes (G4s). *Brca2*-deficient GCPs exhibited decreased replication speed in the presence of the G4-stabilizer pyridostatin. *Pif1* helicase, which resolves G4s during replication, was highly upregulated in tumors, and *Pif1* knockout in primary medulloblastoma tumor cells resulted in increased genome instability upon pyridostatin treatment. These data suggest that G4s may represent sites prone to replication stalling in highly proliferative GCPs and without BRCA2, G4s become a source of genome instability. Tumor cells upregulate G4-resolving helicases to facilitate rapid proliferation through G4s highlighting PIF1 helicase as a potential therapeutic target for treatment of BRCA2-deficient medulloblastomas.

## Significance

This study highlights a specific source of endogenous DNA replication stress, G-quadruplexes (G4s), that can increase genome instability in BRCA2-deficient cerebellar granule cell progenitors (GCPs), leading to medulloblastoma. BRCA2 is necessary for maintenance of replication speed at G4s in GCPs, and its absence allows these structures to become hotspots of increased mutagenesis. The G4-unwinding PIF1 helicase was found to be upregulated in tumor cells as a potential way to cope with G4-related replication stress. Targeting PIF1 may represent a therapeutic strategy for BRCA2-deficient medulloblastomas.

Patients with biallelic pathogenic variants in the *BRCA2* gene belong to Fanconi anemia complementation group D1 (FA-D1) (1). Classically, these patients present with congenital abnormalities and are predisposed to leukemia and embryonal tumors at an early age, although the presence of certain *BRCA2* variants may result in milder disease (1–3). BRCA2 is a large 3,418 amino acid protein that maintains genome stability through its dual roles in promoting homology-directed repair (HDR) of DNA double-strand breaks (DSBs) (4) and protecting stalled replication forks from excessive degradation by nucleases (5).

BRCA2 has several interaction partners that facilitate its critical role in genome stability. BRCA2 mediates RAD51 loading and stable nucleoprotein filament formation on single-stranded DNA (6, 7). The BRCA2 N-terminal domain contains an interaction site for PALB2 to allow for localization to DSBs and stalled forks and for efficient HDR (8). Patients with biallelic *PALB2* variants belong to the FA-N complementation group and have a predisposition to embryonal tumors of childhood such as medulloblastoma, neuroblastoma, and Wilms tumor, similar to FA-D1 patients (9, 10).

Patients in the FA-D1 complementation group develop medulloblastoma (MB) as one of their earliest malignancies, and these tumors are typically classified as Sonic Hedgehog (SHH) subgroup (11). MB arising from BRCA2 deficiency was previously modeled in mice with nervous system-wide *Brca2* mutation targeted to exon 11 that affected cerebellar development and led to medulloblastoma formation (12).

The cell of origin of SHH-MB is the granule cell progenitor (GCP) of the cerebellum (13, 14). These neuronal progenitors proliferate highly in the early postnatal period and give rise to the cerebellar granule cells, the most numerous neuron population in the brain (15). GCPs undergo extensive clonal expansion in response to activation of the SHH signaling pathway in the early postnatal period (16, 17). This is followed by cell cycle exit and migration from the external germinal layer (EGL) of the cerebellum to the inner granule layer and differentiation into multipolar granule neurons (18).

Constitutive SHH activation through loss of PTCH1 in GCPs can lead to MB formation in mouse models through unchecked GCP proliferation (19). Analogously, germline pathogenic *PTCH1* variants associated with Gorlin syndrome lead to medulloblastoma in humans (20). In vitro, activation of SHH signaling by treating GCPs with the N-terminal fragment of SHH (SHH-N) leads to faster DNA replication resulting in DNA damage in S-phase cells and increased origin licensing (21). These findings suggest that GCPs may be especially sensitive to fork stalling at replication-blocking lesions during the proliferative phase of their development. Such stalling could be triggered by DNA lesions, proteins trapped on DNA, depletion of nucleotides required for ongoing DNA synthesis, and unresolved DNA secondary structures such as RNA/DNA hybrids and G-quadruplexes (G4s) that impede the replication machinery (22).

G4s are DNA secondary structures that distort the DNA helix, stall replicative polymerases (23), and are linked to mutations in human cancers (24). G4s may lead to increased DNA breakage in the absence of BRCA2 due to replication stalling (25). G4s can be unwound by specific helicases including werner syndrome RecQ like helicase (WRN), bloom syndrome, RecQ like helicase (BLM), FA complementation group J (FANCJ)/BRCA1 interacting DNA helicase 1 (BRIP1) and Petite Integration Factor 1 (PIF1) as reviewed in ref. 26. In this study, we highlight the intrinsic vulnerability of *Brca2^Δex3-4^* GCPs to replication stalling at G-quadruplex-rich loci that may lead to the mutations that drive medulloblastoma and the role of the PIF1 helicase in promoting tumor maintenance.

## Results

**Fig. 1.**
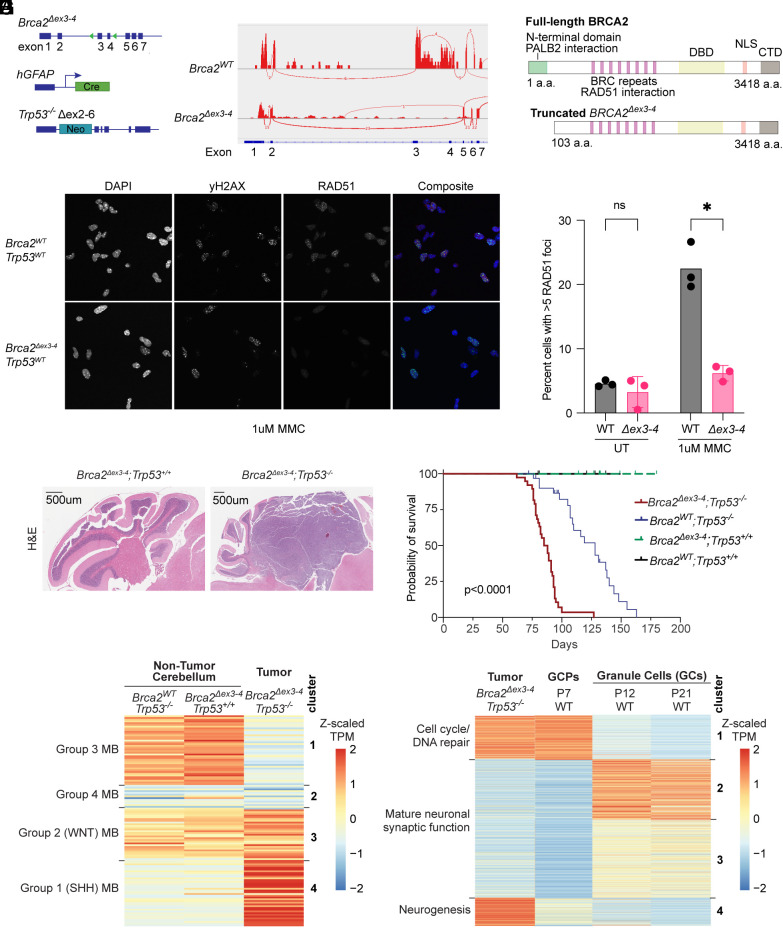
Loss of the PALB2-binding domain of BRCA2 and global loss of TP53 lead to the development of SHH subgroup medulloblastoma. (*A*) Summary of genetic perturbations in the mouse model of medulloblastoma used in the study. *Brca2* with floxed exon 3 to 4, hGFAP-Cre transgene [FVB-Tg(GFAP-cre)25Mes/J], *Trp53* with neo cassette replacing exons 2 to 6 (B6.129S2-Trp53tm1Tyj/J). (*B*) Sashimi plots of splicing in *Brca2^WT^* GCPs and *Brca2^Δex3-4^* tumor demonstrates alternative splicing in *Brca2^Δex3-4^* between exons 2 and 5. (*C*) Diagram of truncated BRCA2 without PALB2-binding domain. (*D*) *Brca2^Δex3-4^*; Trp53+/+ GCPs are deficient in RAD51 foci formation after treatment with 1 μM MMC for 5 h. Representative images shown with DAPI (blue), γH2AX (green), and RAD51 (red). *Brca2^WT^; Trp53^+/+^* GCPs are competent for RAD51 foci formation. (*E*) Percentage of cells with greater than 5 RAD51 foci increases after MMC treatment for *Brca2^WT^* GCPs but not *Brca2^Δex3-4^* GCPs. (*P* = 0.02). (*F*) Hematoxylin and eosin–stained sagittal sections of the mouse cerebellum from *Brca2^WT^; Trp53^−/−^*and *Brca2^Δex3-4^; Trp53^−/−^* mice at postnatal day 90 (P90) demonstrate medulloblastoma formation in the *Brca2^Δex3-4^; Trp53^−/−^*cerebellum. (*G*) Kaplan–Meier survival curve of *Brca2* and *Trp53* mutant animals demonstrates a significant decrease in survival due to medulloblastoma formation in *Brca2^Δex3-4^; Trp53^−/−^*animals. *Brca2^WT^; Trp53^−/−^* did not form medulloblastomas but had decreased survival, presumably due to other malignancies such as lymphomas. (*H*) Heatmap of normalized gene expression of medulloblastoma subgroup hallmark genes from RNA sequencing of bulk tumor and age-matched adult normal cerebellum. (*I*) Heatmap of normalized gene expression from bulk tumor RNAseq as well as differentially expressed genes in proliferating GCPs compared to postmitotic granule cells (31).

Loss of the PALB2-binding domain of BRCA2 coupled with TP53 loss leads to the development of SHH subgroup medulloblastoma. To identify endogenous lesions that may lead to MB development in patients with BRCA2 deficiency, we coupled the *Brca2*^Δ^*^ex3-4^* mouse model (27) with global *Trp53* knockout (28). Conditional deletion of exons 3 and 4 of *Brca2* leads to breast carcinoma development when Cre expression is driven by the *Wap* promoter (27). It also results in replication stress, mitotic abnormalities, and G1 arrest in mammary epithelial cells (29). We expressed Cre under the control of human glial fibrillary acidic protein (*hGFAP*) promoter, leading to a conditional loss of exons 3 and 4 of *Brca2* in the developing mouse central nervous system (CNS) (Fig. 1*A*). We observed the loss of exons 3 and 4 using RNA sequencing and whole-genome sequencing of *Brca2*^Δ^*^ex3-4^* tumors and GCPs (Fig. 1*B*). However, the expression of BRCA2 protein appeared unchanged as assessed by western blotting using an antibody recognizing amino acids 1651 to 1812 (*SI Appendix*, Fig. S1*A*). In this model, alternative splicing between exons 2 and 5 (Fig. 1*B*) allows for expression of an alternate in-frame methionine in exon 7 leading to loss of 103 amino acids of the N terminus, a change that is not visible on a western blot (Fig. 1*C*). The resulting mutation was confirmed through WGS of the BRCA2 region in cerebellar tissue, demonstrating deletion of the region spanning exons 3 and 4 (*SI Appendix*, Fig. S1*B*). Mutation of the BRCA2 N terminus disrupts its PALB2-binding domain (29) and can lead to abrogated recruitment of BRCA2 to chromatin and diminished RAD51 filament formation (30). Indeed, *Brca2^WT^; Trp53^+/+^* GCPs but not *Brca2^Δex3-4^; Trp53^+/+^* GCPs induced RAD51 foci after treatment with mitomycin C (MMC) (Fig. 1 *D* and *E*).

When *Brca2*^Δ^*^ex3-4^* was coupled with global *Trp53* knockout, mice formed medulloblastomas with complete penetrance and exhibited a significant decrease in survival due to the tumor burden of double mutants (Fig. 1 *F* and *G*) in contrast to *Brca2^WT^; Trp53^−/−^* and *Brca2^Δex3-4^; Trp53^+/+^* animals, which did not develop MBs. This finding is consistent with MBs only forming on a *Trp53^−/−^* background in the majority of genetically engineered mouse models of medulloblastoma (32). Although they did not form MBs, *Brca2^WT^; Trp53^−/−^* animals experienced increased mortality presumably due to malignancies associated with Trp53 loss, such as lymphomas (28). MBs that formed in *Brca2^Δex3-4^; Trp53^−/−^* animals were highly proliferative and contained markers of DNA damage as indicated by the strong Ki67 staining and scattered γH2AX staining *SI Appendix*, Fig. S1*C*). *Brca2^Δex3-4^; Trp53^+/+^* animals exhibited mild cerebellar hypoplasia at postnatal day 7, the developmental time at which GCPs are highly proliferative in the EGL (*SI Appendix*, Fig. S1*D*). Tumors were easily identifiable by their 5-ethynyl-2′-deoxyuridine (EdU) staining even at postnatal day 50 (*SI Appendix*, Fig. S1*E*).

Guided by the previously identified expression changes in clinical subgroups of MB (33, 34) (Dataset S1), we performed hierarchical clustering of tumor and age-matched control cerebellar expression data (Fig. 1*H*). *Brca2*^Δ^*^ex3-4^*; *Trp53*^−/−^ MBs highly expressed genes associated with the SHH subgroup, including those involved in activated Hedgehog signaling and the cell cycle (Cluster 4, Dataset S1, primary data can be found in ref. 35) and downregulated genes associated with Group 3 and 4 MB including those associated with G-protein-coupled receptor signaling, cell migration, and axon guidance (Clusters 1 and 2, Dataset S1). We conclude that *Brca2*^Δ^*^ex3-4^*; *Trp53*^−/−^ MBs have a gene expression profile most like clinical SHH MB cases.

Previous studies have pointed to the cerebellar GCP as the cell of origin for SHH MB (13, 14). To assess the similarity between the tumor’s gene expression profile and that of GCPs, we performed hierarchical clustering using a list of genes found to be differentially expressed (*P*adj < 0.01) in proliferating Math1-positive P7 GCPs compared to postmitotic NeuroD1-positive GCs (31) (Dataset S2).

Cluster 1 represents genes highly expressed in both proliferating, P7 GCPs, and tumors. These genes are associated with pathways of mitotic cell cycle, DNA replication, and homologous recombination and include *Cdk1* that regulates mitotic entry and *CyclinD2* that regulates G1 progression (Fig. 1*I*). Other genes within cluster 1 are *Math1/Atoh1*, *Myc*, and *Pif1*. Clusters 2 and 3 were enriched in GCs at P12 and P21 and not expressed in tumors, and these clusters both contained genes associated with neurotransmitter signaling. Cluster 4 was the most tumor-enriched gene expression cluster, which diverged from non-tumor-proliferating GCPs. This cluster included genes related to glial-guided migration, genes with roles in neurite outgrowth, and genes implicated in the cellular response to fibroblast growth factors. Overall, the gene expression analysis reveals that *Brca2*-deficient MBs have a gene expression program similar to P7 GCPs but also acquire additional expression changes that may promote tumor growth through pathways divergent from those active during normal GCP proliferation.

### *Brca2*-Mutant Medulloblastoma Is Driven by *Ptch1* Loss and Characterized by Mutations Overlapping G-Quadruplexes.

**Fig. 2.**
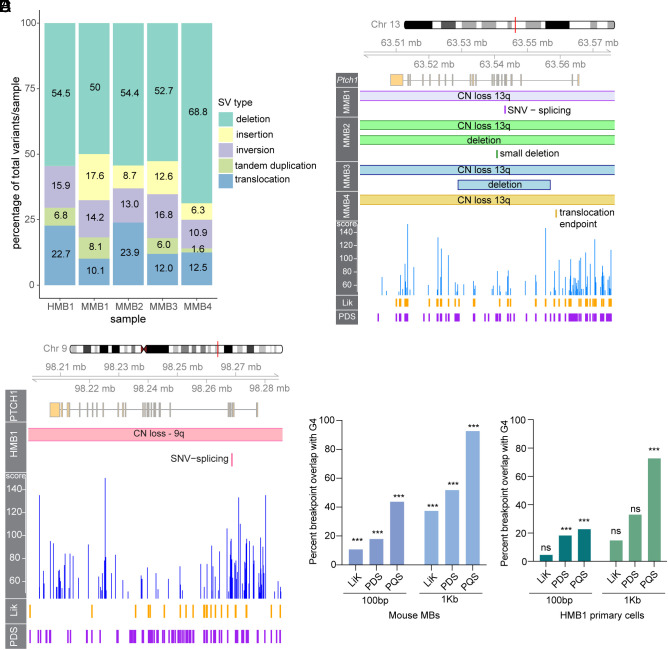
*Brca2* mutant medulloblastomas are driven by mutations that overlap with G-quadruplexes. (*A*) Percentages of SV class per sample of HMB1 and mouse MB samples 1-4 (MMB1-4). (*B*) Genome visualization (Gviz) plot of mouse *Ptch1* depicts CN loss of chromosome 13q and secondary mutation in each mouse MB sample in this G4-dense gene. (*C*) Gviz plot of HMB1 demonstrates CN loss of chromosome 9q and splicing SNV that inactivates *PTCH1*. (*D*) Statistical overlap test performed in regioneR (37) of tumor CNV breakpoints and G4-forming sequences from experimental and computational datasets (38, 39) demonstrates a significant overlap (*P* = 0.001) of breakpoints and G4s when compared to random shuffling of breakpoint windows on the mouse genome. (*E*) Percentage overlap with PQSfinder G4s of each mutation type at 100 bp breakpoints and 1 kb breakpoints.

To assess the mutational landscape of *Brca2^Δex3-4^* MB, we performed whole-genome sequencing on DNA isolated from four mouse medulloblastomas (MMB1-4) at 60× genome coverage and matched nontumor forebrain tissue at 30× genome coverage. After filtering germline variants using the sequenced matched forebrain, we identified 643 variants across four tumors. The largest proportion of variants in the *Brca2^Δex3-4^; Trp53^−/−^* mouse MBs were small deletions (Fig. 2*A*, *SI Appendix*, Fig. S2*A*, and Dataset S3, primary data can be found in ref. 36). In addition, we detected structural variants (SVs) such as large deletions, translocations, inversions, and tandem-duplications that were highly variable in size, between 379 bp and 98 Mbp (*SI Appendix*, Fig. S2*B*). A parallel analysis of copy number variation (CNV) identified 107 regions of copy number gain and 112 regions of copy number loss (*SI Appendix*, Fig. S2*C* and Dataset S3).

We also generated a human MB primary cell line 1 (HMB1) from an FA-D1 pediatric patient with germline compound heterozygous variants in *BRCA2* (c.9097_9098insT and c.9302 T > G) who was diagnosed with MB at the age of 3 years. Clinically, this FA-D1 MB was determined to have SHH activation through increased YAP1 and GAB1 staining as well as p53 deficiency by IHC. *TP53* variants detected through sequencing of HMB1 included a copy number loss of one allele and a pathogenic SNV (c.412G > C) in the second allele. The HMB1 primary cells expressed GCP markers including PAX6 and TAG1 (*SI Appendix*, Fig. S2*D*). We sequenced the DNA isolated from this human primary MB cell line at 60× genome coverage.

The HMB1 sample contained 44 SVs, 27 regions of copy number gain, and 47 regions of copy number loss (*SI Appendix*, Fig. S2 *E*–*G* and Dataset S4). These variants were determined after filtering using a panel of human controls due to the lack of normal tissue from this FA-D1 patient to perform germline filtering. Due to the inability to appropriately filter germline indel variants in HMB1, we omitted small indels from this analysis. SVs from the HMB1 line were enriched for deletions with a similar proportion as in the MMB samples (Fig. 2*A*).

Few recurrent mutations were identified across the murine tumor samples, with the exception of mutations in *Ptch1* that were detected in all samples (Fig. 2*B*). Copy number loss of chromosome 13q where *Ptch1* is located was coupled in each sample with an inactivating mutation of the other allele of *Ptch1*. Mutations of the second allele of *Ptch1* included a single nucleotide variant predicted to lead to abnormal splicing and an early stop codon in MMB1, a large deletion and a smaller deletion in MMB2, a large deletion in MMB3, and a translocation upstream of *Ptch1* to chromosome 5 in MMB4. The human BRCA2-deficient MB carried *PTCH1* mutations following the pattern detected in mouse *Brca2^Δex3-4^* tumors. It contained a loss of one copy of chromosome 9q, a common copy number alteration in pediatric SHH MB, coupled with an inactivating SNV leading to alternative splicing and truncation of *PTCH1* (Fig. 2*C*).

The *PTCH1* gene has previously been reported to have a high abundance of G-quadruplex forming sequences (40). We utilized the putative quadruplex sequence (PQS) detection package pqsfinder to identify the potential G4 sequences in mouse *Ptch1* (Fig. 2*B*). pqsfinder computes a G4 score that indicates the predicted stability of each G4 and therefore its propensity to form. We found that *Ptch1* has in total 443 G4s, 88 of which are above score 50, indicating high predicted stability. Many of the somatic mutations in *Ptch1* occur between two peaks of high G4 stability coupled with high G4 density of overlapping putative G4s (Fig. 2 *B* and *C*).

Following the observation of a high G4 burden in the *Ptch1* locus, we hypothesized that other tumor variant breakpoints would significantly overlap with putative G4 loci in MBs. To test this hypothesis, we performed overlap testing of 100 bp or 1 Kb ranges around the breakpoint ends of the detected SVs and indels in the four mouse tumors, against pqsfinder-derived putative G4s (*SI Appendix*, Fig. S3 *A*–*D*), as well as in two experimental datasets generated from stabilizing DNA libraries from mouse fibroblasts with lithium and potassium (LiK) or the G4-trapping drug pyridostatin (PDS) and performing mismatch analysis to identify where the sequencing polymerase stalled at stabilized G4s (38). For both the 100 bp and 1 Kb breakpoint windows, we found high overlap of breakpoints with the PDS-stabilized or computationally predicted G4s and a more moderate overlap with the LiK dataset (Fig. 2*D*). Permutation testing with regioneR revealed that the overlaps were significant (*P* = 0.001) compared to random shuffling of breakpoints in the genome (*SI Appendix*, Fig. S4 *A*–*F*) at both the 100 bp and 1 Kb breakpoint window sizes. We did not observe a particular bias for any class of mutation with G4 overlap (*SI Appendix*, Fig. S3 *A* and *B*). This indicates that the mutagenic repair of DNA breaks at potential G-quadruplex DNA may lead to diverse mutation outcomes observed in tumor samples.

We next performed overlap analysis using SV breakpoints from HMB1 utilizing pqsfinder-derived G4s identified from the human genome, and LiK and PDS-stabilized datasets detected from human HEK-293T cells (38). Although there were only 88 SV breakpoints found in HMB1, we identified significant overlap with PQS G4s at both 100 bp and 1 Kb breakpoint windows from regioneR statistical tests (Fig. 2*E* and *SI Appendix*, Fig. S4 *G*–*L*). As in mouse samples, we did not observe a bias for any SV type in the HMB1 sample (*SI Appendix*, Fig. S3 *E*–*G*. These data indicate that the presence of G4s may influence DNA breakage in human SHH-MB samples and lead to subsequent mutagenesis in G4-dense loci. The absence of BRCA2 may contribute to DSB formation at G4s or prevent proper repair leading to SV formation (*See discussion*).

### GCPs Are Highly Replicative under SHH Pathway Activation and Sensitive to G4 Trapping by Pyridostatin.

**Fig. 3.**
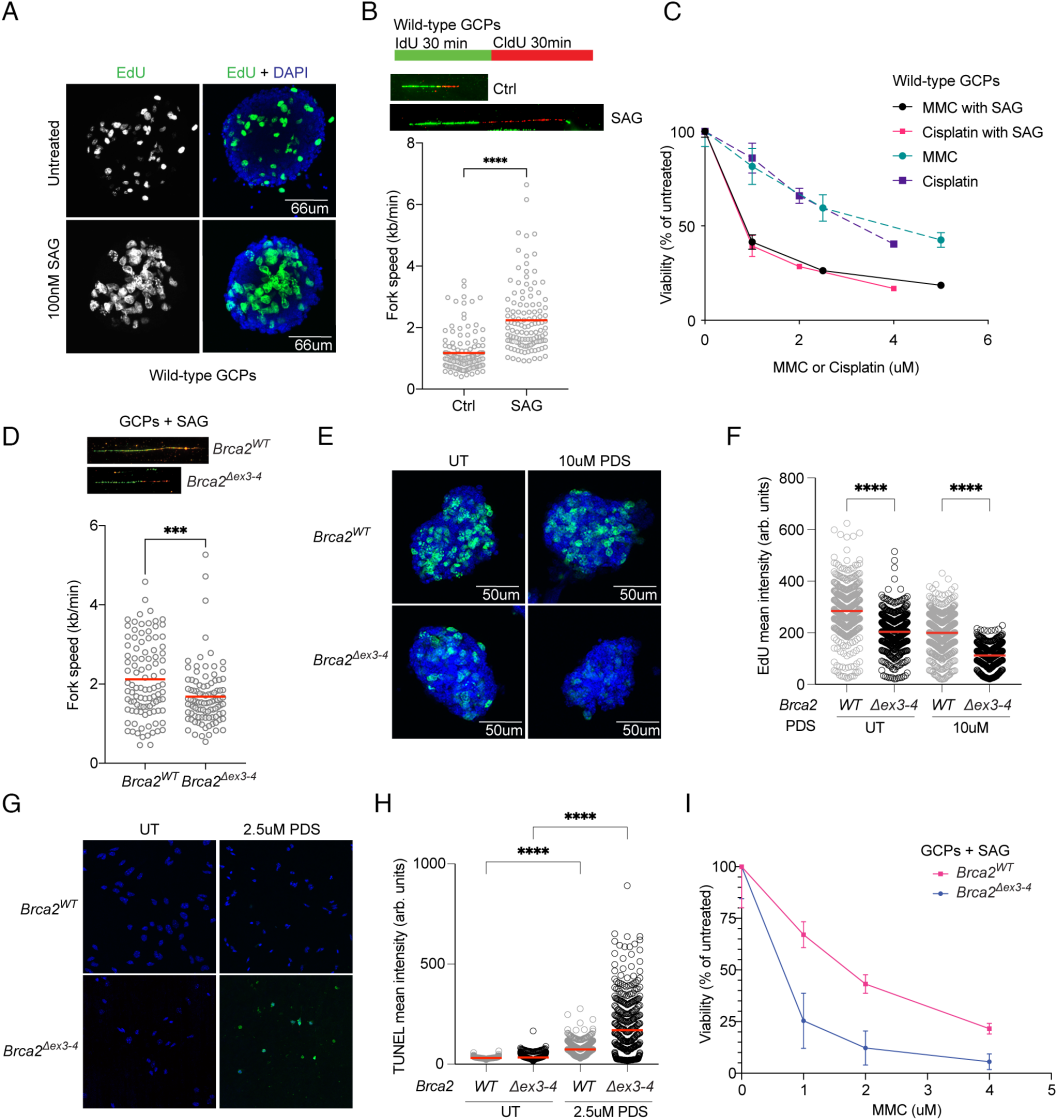
GCPs are highly replicative under SHH pathway activation and sensitive to G4 trapping by pyridostatin. (*A*) GCPs isolated from P7 wild-type C57BL.6 animals were cultured with or without 100 nM SAG for 24 h as proliferating reaggregates and pulsed with EdU for 30 min. (*B*) SAG treatment for 24 h additionally leads to faster replication fork speeds of 2.3 kb/min on average, compared to 1.17 kb/min in non-SAG-treated samples. (*C*) CellTiter-Glo viability assay on wild-type GCPs treated with MMC and Cisplatin with or without SAG demonstrates a decrease in viability with SAG and treatment with a DNA crosslink-inducing agents. Error bars are ± SD. (*D*) Representative confocal images of GCP reaggregates treated with 100 nM SAG and 10 μM PDS for 2 h prior to 10 min EdU pulse. DAPI nuclear staining in blue and EdU staining in green. (*E*) Mean nuclear intensity of EdU incorporation in reaggregates demonstrates a decrease in DNA replication level in *Brca2^Δex3-4^; Trp53^+/+^* GCPs after 2 h of 10 μM PDS treatment. 300 cells scored for each sample per replicate of three biological replicates (*P* < 0.0001). (*F*) DNA combing of *Brca2^Δex3-4^; Trp53^+/+^*and *Brca2^WT^; Trp53^+/+^* GCPs in the presence of SAG. *Brca2^Δex3-4^* GCPs demonstrate a mildly decreased fork speed of 1.7 kb/min compared to Brca2WT GCPs at 2.2 kb/min (*P* = 0.0005). (*G*) TUNEL staining for apoptosis in GCPs plated down immediately after isolation on PDL/Matrigel-coated coverslips demonstrates an increase in apoptosis of *Brca2^Δex3-4^; Trp53^+/+^* GCPs. DAPI nuclear staining in blue and TUNEL staining in green. 300 cells scored for each sample per replicate of three biological replicates (*P* < 0.0001). (*H*) Mean intensity of TUNEL signal in individual adherent GCPs quantified in Imaris. Quantification demonstrates an increase in TUNEL-positive *Brca2^Δex3-4^; Trp53^+/+^* GCPs after PDS treatment (*P* < 0.0001). (*I*) Cell viability measured with the CellTiter-Glo reagent demonstrates that survival of *Brca2^Δex3-4^; Trp53^+/+^* GCPs decreases more in the presence of MMC compared to *Brca2^WT^; Trp^53+/+^* GCPs. Error bars represent ± SD.

The biology of mouse GCPs can be assessed ex vivo by culturing GCPs as proliferative reaggregates in suspension or as differentiated granule neurons in monolayer cultures plated on poly-D-lysine/Matrigel coverslips. GCPs have been shown to exhibit replication stress upon SHH pathway activation by treatment with the N-terminal fragment of SHH (21). We confirmed this observation by treating wild-type GCPs with the Smoothened agonist (SAG) that leads to constitutive SHH pathway activation. The overall number of EdU-positive GCPs grown as reaggregates increased with SAG treatment (Fig. 3*A*). To observe the influence of SAG on individual replication forks, we performed DNA combing on wild-type GCPs with sequential pulses of halogenated nucleotide analogs IdU and CldU (Fig. 3*B*). SAG treatment increased the average fork speed of wild-type GCPs from 1.2 kb/min in control cells to 2.3 kb/min, the highest of published fork velocity as measured by DNA combing across different cell lines (41), which range from 0.7 kb/min to 2 kb/min.

We analyzed whether increased fork speed following SAG treatment led to greater DNA damage by examining γH2AX staining in proliferating GCPs. γH2AX intensity was significantly increased in SAG-treated GCPs, and this DNA damage was exacerbated further when cells were treated with the DNA crosslinking drug cisplatin which stalls replication (*SI Appendix*, Fig. S5 *A* and *B*). The observed increase in DNA damage also correlated with lower viability when wild-type GCPs were treated with SAG and crosslinking agents MMC or cisplatin, compared to DNA crosslinking drugs alone (Fig. 3*C*). These findings indicate that SHH pathway activation by SAG drives increased replication speeds that render cells sensitive to replication stress. In vivo, GCPs require SHH secreted from neighboring Purkinje neurons (16) to proliferate and expand to the most numerous single neuron in the brain (15). This suggests that during the massive proliferation that underlies neurogenesis, GCPs may exhibit an intrinsic vulnerability to replication-blocking DNA lesions that could be exacerbated upon SHH stimulation.

To test whether BRCA2 is required to promote genome stability in GCPs experiencing replication stress during the proliferative phase of cerebellar development, we performed DNA combing in *Brca2^Δex3-4^* GCPs. We found that fork speed decreased significantly in *Brca2^Δex3-4^; Trp53^+/+^* GCPs treated with SAG from 2.2 kb/min to 1.7 kb/min (Fig. 3*D*). This slower fork speed could be due to increased stalling at genomic lesions in fast replicating cells when BRCA2 is absent. Based on our findings in tumors, G-quadruplexes could be the source of replication stress, thus we assessed whether G4s block replication in *Brca2^Δex3-4^* GCPs by treating proliferating GCP reaggregates with pyridostatin (PDS), a G4 stabilizing chemical (Fig. 3*E*). Incorporation of EdU, a marker of replication, significantly decreased in *Brca2^Δex3-4^* GCPs after a 2-h treatment with high-dose PDS (Fig. 3*F*). PDS treatment additionally decreases the EdU mean intensity of *Brca2^WT^* GCPs, indicating that G4-stabilization serves as a replication block, perhaps temporarily in wild-type GCPs.

Cell viability downstream of replication stalling, as assessed by TUNEL staining for apoptosis following PDS treatment was decreased in *Brca2^Δex3-4^; Trp53^+/+^*, but not in *Brca2^WT^; Trp53^+/+^* GCPs when treated with PDS (Fig. 3 *G* and *H*). These data indicate that BRCA2 deficiency sensitizes GCPs to cell death after treatment with G4-stabilizing drugs, perhaps due to an increase in DNA breaks at unprotected stalled forks or due to deficiency of DSB repair. As predicted, treatment with DNA crosslinking agents MMC and SAG also reduced *Brca2^Δex3-4^; Trp53^+/+^* GCP viability (Fig. 3*I*), as expected due to BRCA2 function in interstrand crosslink repair.

### The PIF1 Helicase Protects *Brca2*^Δ^*^ex3-4^* Medulloblastoma Cells against Genomic Instability at G-Quadruplexes.

**Fig. 4.**
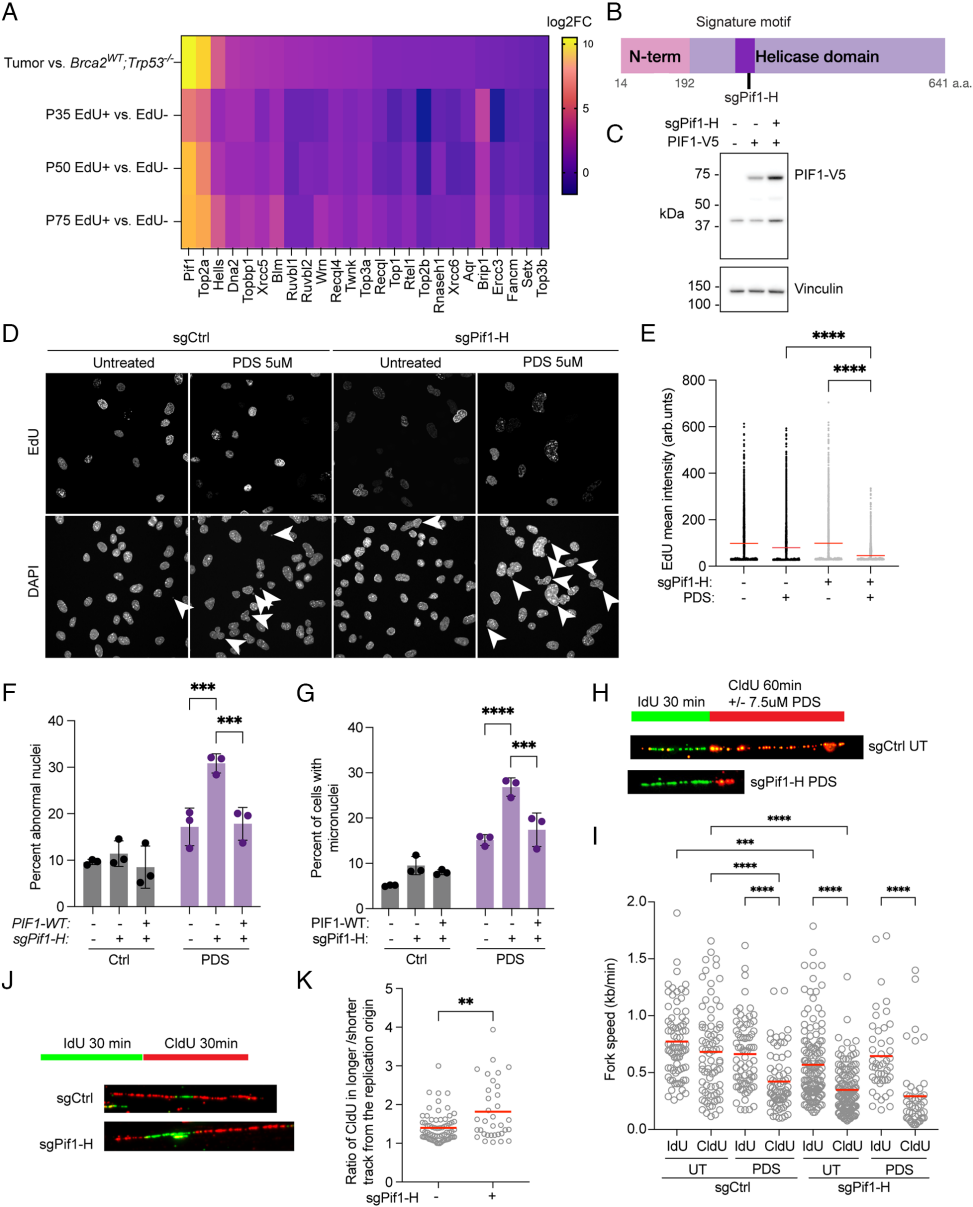
The PIF1 helicase protects *Brca2^Δex3-4^* medulloblastoma cells against genomic instability at G-quadruplexes. (*A*) Heatmap of log2 fold change (log2FC) values from differential gene expression datasets demonstrates *Pif1* and *Top2a* upregulation in *Brca2^Δex3-4^* tumor compared to nontumor Brca2WT cerebellum. (*B*) Schematic of PIF1 protein indicating the domain targeted by the *sgPif1-H* in primary *Brca2^Δex3-4^* tumor cells. (*C*) Immunoblot with an antibody recognizing V5-tagged PIF1 and the loading control (Vinculin) demonstrates an increase in PIF1 expression when endogenous *Pif1* is knocked out. (*D*) Representative images of sgNeg or *sgPif1-H* KO cells stained with DAPI and EdU after 48 h of treatment with and without 5 μM PDS. White arrows point to abnormal nuclei and micronuclei, which increase in bulk *sgPif1-H* KO with PDS treatment. (*E*) Mean nuclear intensity of EdU demonstrates a decrease in the level of DNA replication in *sgPif1-H* MB cells (*P* < 0.0001). (*F*) Abnormal nuclei percentage as determined with a Imaris machine-learning pipeline. 300 nuclei scored per sample in each biological replicate of three biological replicates (*P* = 0.0006, *P* = 0.0009). (*G*) Micronuclei hand-counted per 300 DAPI-stained cells in each sample across three biological replicates (*P* = 0.0001, *P* = 0.0009). sgNeg and *sgPif1-H* KO cells demonstrate increased micronuclei after PDS treatment, with a greater increase in *sgPif1-H* KO cells. (*H*) DNA combing scheme for MB cells where cells are pulsed with IdU for 30 min, followed by a 60 min CldU pulse with or without PDS to assess fork stalling after G4 stabilization. (*I*) Fork speed quantification reveals a decrease in fork speed in *sgCtrl* MB cells with PDS treatment, untreated *sgPif1-H* cells as replication progresses in the second CldU label, and *sgPif1-H* cells treated with PDS (*P* < 0.0001). (*J*) Schematic of labeling to assess replication speed asymmetry. (*K*) Combing of MB1 cells demonstrating greater origin asymmetry when *Pif1-H* guide was used (*P* = 0.004). UT = untreated.

The potential importance of G-quadruplex resolution in the BRCA2-deficient medulloblastoma was further highlighted when we assessed gene expression changes in tumor compared to age-matched normal cerebellum and identified that the 5’-3’ DNA helicase PIF1, which has ability to bind to G4s (42) and promotes processivity through G4s (43) was one of the most upregulated genes in tumors (Fig. 4*A*). Topoisomerase IIA (*Top2A*), which functions in releasing DNA supercoiling during replication, was also highly upregulated in tumor samples, highlighting the particular importance of DNA processing during replication in MBs. Notably, other G4 helicases such as *Blm*, *Wrn*, and *Brip1/Fancj* were also expressed but not differentially expressed in tumor versus normal cerebellum. RNAseq from samples serially harvested across tumor development, by sorting EdU-pulsed cells from the adult, postmitotic cerebellum at P35, P50, and P75, revealed that *Pif1* upregulation occurred as early as P35, which coincided with early tumor development (Fig. 4*A* and *SI Appendix*, Fig. S1*D*).

Beyond its ability to unwind G-quadruplex DNA, DNA/RNA hybrids, and forked DNA (44), PIF1 has been shown to facilitate genome stability of G4-rich DNA in yeast (43) and RAS-transformed human fibroblasts (45). In *Drosophila* embryos, PIF1 was found to be necessary for overcoming replication stress and its loss was synthetically lethal with BRCA2 mutation (46).

To assess the role of PIF1 in *Brca2^Δex3-4^* MB, we utilized primary cells isolated from mouse MBs. These cells had neuron-like morphology, remained proliferative in culture, and expressed markers typical of *Brca2^Δex3-4^* tumors such as *Tag1* and *Pax6* (*SI Appendix*, Fig. S6*A*). We targeted *Pif1* with a CRISPR/Cas9 sgRNA directed at the signature motif within the helicase domain required for PIF1 activity (47) (*sgPif1-H*). Prior to CRISPR-directed cutting, primary MB cells were complemented with C-terminally V5-tagged mouse *Pif1* cDNA with a synonymous PAM-site mutation making it resistant to CRISPR guide-directed cleavage (Fig. 4*B*). The gRNA produced high bulk cutting efficiency within *Pif1* (*SI Appendix*, Table S1) and the V5-tagged *Pif1* expression was maintained after cutting using *sgPif1-H* (Fig. 4*C*).

Single-cell clones with frameshift mutations in the PIF1 helicase domain, exhibited significant increases of large, abnormal nuclei and micronuclei (*SI Appendix*, Fig. S6 *B*–*D*). These phenotypes of genomic instability were exacerbated further when clones were complemented with a helicase dead mutant of PIF1 (E316Q) (*SI Appendix*, Fig. S6 *E*–*G*) (48). Bulk targeting with *sgPif1-H* resulted in increased levels of aberrant nuclei that were further increased following PDS treatment but decreased with complementation using PAM-resistant WT *Pif1* (Fig. 4 *D* and *F*). Similarly, micronuclei were found to be significantly more frequent in PIF1-deficient cells treated with PDS (Fig. 4*G*). They also exhibited decreased EdU incorporation, indicating slowed replication rates consistent with replication stress (Fig. 4*E*).

We then examined the effects of PDS on individual replication forks of PIF1-deficient MB cells by DNA combing. PIF1-deficient MB cells had slower rates of replication than control cells, with particularly decreased fork speed during the second pulse, which suggests that these forks encounter stalling lesions (Fig. 4 *H* and *I*). Further support for heightened fork stalling in these cells came from analysis of replication fork asymmetry, which was significantly increased in *Pif1*-KO cells (Fig. 4 *J* and *K*). PIF1-competent MB cells also demonstrated a decrease in fork speed after PDS treatment, which indicates that endogenously expressed PIF1 can be overwhelmed by high levels of stabilized G4s. These findings indicate that replication through G4 DNA is facilitated by the PIF1 helicase in primary MB cells and suggest that PIF1 upregulation is essential to support replication through potential G4-forming sequence as *Brca2^Δex3-4^* MB cells proliferate. Targeting PIF1 by chemical inhibition or genetic modification in BRCA2-deficient MBs could allow for selective killing of tumor cells, sparing surrounding normal cerebellum that lacks *Pif1* expression.

## Discussion

This study aimed to identify endogenous causes of vulnerability of a highly proliferative developmental cell population, the cerebellar GCP, in the setting of BRCA2 deficiency. Using in vitro and in vivo approaches, we have identified G-rich DNA capable of creating G4s, as being a challenge for BRCA2-deficient GCPs. We determined a significant association of SV and indel breakpoints with putative G4s in the spontaneously formed medulloblastomas of the *Brca2^Δex3-4^; Trp53^−/−^* mouse model. These included inactivating mutations in the G4-dense gene *Ptch1,* a most likely primary driver of tumorigenesis in all MBs formed in our model, which is consistent with previous studies of a BRCA2 model of MB (49) and genomic studies of SHH-group MB (11) that also identified *PTCH1* mutation as recurrent in pediatric SHH tumors.

Prior studies suggest that different tissues and cells may display vulnerability to different endogenous sources of genome instability (50, 51). It is unclear why the GCPs would be particularly vulnerable to instability at G4s. Both replication speed and DNA damage increase when GCPs are stimulated by SHH (21). GCPs therefore may be particularly vulnerable to fork stalling because of the demands of their postnatal expansion. Under those conditions, BRCA2 plays an integral role in preventing genomic instability at G4s in GCPs, averting medulloblastoma development. However, it remains to be determined whether the essential function of BRCA2 at G4-rich regions is to protect stalled forks allowing for its nonmutagenic processing and prevention of replication fork collapse, or alternatively, the HDR of the DNA damage that occurs when such collapse occurs at the G4s. This instability at G4-forming sequence and resulting tumorigenesis is permissible under conditions of *Trp53* deficiency, whereas growth arrest occurs in *Trp53*-proficient cells, as observed with cerebellar hypoplasia in *Brca2*-mutant animals with wild-type *Trp53* (12). The p53 status of cells has implications for treatment options as these cells will continue to proliferate even with increased genomic instability.

Given that embryonal tumors in patients with biallelic pathogenic variants of BRCA2 include neuroblastoma and Wilms tumors, it is tempting to speculate that the essential function of BRCA2 in prevention of these two blue cell tumors will also include BRCA2 function at G4-quadruplexes. This hypothesis will have to be tested in future studies. The connection of G4s and BRCA2 may also be important in other settings of BRCA1/2 deficiency, as the stabilization of G4 DNA by PDS leads to cell death in BRCA1/2-deficient cells (52) and in patient derived xenografts (25). More broadly, CNV breakpoints in human cancers were previously found proximal to G4s (24), with G4-forming sequences located downstream of CNV loci potentially causing leading strand replication stalling and DNA breakage leading to mutagenesis.

G-quadruplexes may not be the only lesions that are being tended by BRCA2 in the context of GCPs. Reactive aldehydes, including formaldehyde and acetaldehyde, are formed during cellular metabolism and are detoxified by the enzymes ALDH2 and ADH5 (50). High levels of reactive aldehydes overwhelm the detoxification capacity of ALDH2/ADH5 and result in DNA interstrand crosslinks (ICLs) that require functional Fanconi anemia pathway for repair (53). Given the high metabolic demands of postnatal GCP development, the impact of aldehyde accumulation on tumorigenesis in BRCA2-deficient GCPs should be considered.

BRCA2 has also been shown to regulate the accumulation of RNA/DNA hybrids or R-loops. It potentially does so by regulating promoter-proximal pausing through interaction with RNA polymerase II (54), through interaction with the TREX-2 mRNA export complex (55), and during replication by interacting with the transcription regulator ZFP281 (56). R-loops were also observed to increase in breast cancer cells following estrogen treatment which may contribute to DNA replication stress and genomic rearrangements at R-loop-forming loci (57). Future studies should examine the R-loop burden and localization specifically in GCPs to determine whether they may contribute to their genomic instability if BRCA2 is absent. As the G-quadruplexes are often colocalized with R-loops, especially at G-rich regions that experience negative supercoiling and are actively transcribed (58), future G4 mapping in combination with R-loop mapping in GCPs could highlight more specific regions of vulnerability that would also consider the transcriptional landscape of GCPs.

Analysis of the transcriptome of medulloblastomas that developed in *Brca2^Δex3-4^; Trp53^−/−^* mouse model hinted at the importance of *Pif1* in tumor cells. We found that *Pif1* was expressed both in tumor cells and proliferating GCPs but was not expressed in the normal adult cerebellum. This finding nominates PIF1 as a potential therapeutic target in BRCA2-deficient MB. PIF1 loss led to decreased replication speed in otherwise unperturbed primary tumor cells, indicating that loss or inhibition of this helicase may stall replication forks at lesions such as G4s and could reduce proliferation of tumor cells. However, potentially due to the *Trp53*^*−/−*^ status of MB cells leading to high levels of genomic instability while evading apoptosis, we did not observe bulk decreases in tumor cell viability, indicating that PIF1 inhibition may need to be coupled with another therapeutic, such as etoposide or PARP inhibition to lead to tumor cell death.

PIF1 overexpression has been associated with several other cancer types including testicular, ovarian, and breast cancers, which express PIF1 at high levels (59), as well as cervical cancer (60) and pancreatic cancer (61). High PIF1 expression was additionally found to be correlated with reduced survival outcomes in neuroblastoma patients (62), indicating that the role of PIF1 in G4 biology may be relevant in other cancers. A recent screen of human PIF1 unwinding inhibitors identified 4-phenylthiazol-2-amine molecules as the first potential inhibitors of hPIF1 helicase functionality (63), opening up the possibility for studying the therapeutic potential of PIF1 inhibition in cellular models of cancer with a targeted small molecule inhibitor.

Studies in model organisms have also shown the importance of G4 resolution in maintaining genome stability. In *Caenorhabditis elegans*, loss of the *dog1/FANCJ* G4-unwinding helicase led to both small deletions and more complex rearrangements upstream of G4 loci (64). FANCJ was shown to directly resolve G4s and prevent replication stalling that was enhanced when G4s were stabilized (65). It remains to be tested whether PIF1 and FANCJ act redundantly to prevent instability at G4s in GCPs and MB cells.

To conclude, our study suggests that BRCA2 and G4 helicases work in concert in GCPs to prevent the accumulation of mutations at potential G4-forming loci in this highly proliferative neuronal progenitor population that has an intrinsic vulnerability to DNA damage during replication. BRCA2-deficient MB cells were also shown to upregulate G4 helicases to foster tumor cell proliferation.

## Materials and Methods

### Mouse Husbandry and Tumor Monitoring.

All mouse experiments were approved by the Rockefeller University Institutional Animal Care and Use Committee (IACUC protocol #23039-H). *Brca2^Δex3-4^* mice (a gift from Maria Jasin, Memorial Sloan Kettering Cancer Center, New York, NY) were crossed with *Trp53^+/−^* B6/129S mice (B6.129S2-Trp53tm1Tyj/J, Jackson Laboratories) and *hGFAP-Cre* mice [FVB-Tg(GFAP-cre)25Mes/J, Jackson Laboratories] to generate *Brca2^Δex3-4^* in the CNS coupled with global *Trp53* knockout. All mice were monitored for tumor formation or humane endpoints. Mice were genotyped using primers in *SI Appendix*, Table S2.

### Human Studies and Isolation of Primary Medulloblastoma Cells.

Participant F89P1 with biallelic BRCA2 was entered into the Rockefeller University International Fanconi Anemia Registry under protocol number AAU-0112 (Smogorzewska PI) approved by the Rockefeller University Institutional Review Board. The informed consent was obtained by the PI of AAU-0112 before resection of the medulloblastoma. Primary medulloblastoma tumor cells were isolated and grown to create human medulloblastoma tumor cell line HMB1, as previously described in ref. 66 and is further described in *SI Appendix*, *Supplemental Materials and Methods*.

### Isolation and Culture of Primary GCPs.

GCPs were isolated at P7 from C57BL/6 (strain #000664, Jackson Laboratories) wild-type or *hGFAP-Cre; Brca2^Δex3-4^; Trp53* mutant animals as previously described (67). Plated GCPs were cultured on coated glass coverslips and treated with 100 nM SAG (Cayman Chemical, #11914) for 3 h or 12 h as well as pyridostatin (MedChemExpress #HY-15176A) at 2.5 μM, 5 μM, or 10 μM. EdU (ThermoFisher #E10187) was added at 10 μM 30 min prior to the end of each timepoint. Proliferating GCPs were cultured in suspension and allowed to reaggregate for 16 h prior to treatment (*SI Appendix*, *Supplemental Materials and Methods*).

### Generation of Pif1-KO MB Cells.

CRISPR RNP cutting was performed with Cas9 complexed with assembled sgRNAs targeting mouse *Pif1* and delivered via Amaxa 4D nucleofection (Lonza #V4XP-3032). Single cell clones with homozygous early truncation mutations in *Pif1* were generated through serial dilution of bulk CRISPR cells and subsequent selection of wells with single colonies for further expansion. Sanger sequencing of the *sgPif1-H* surrounding region and Synthego ICE analysis of CRISPR-generated mutations (https://ice.editco.bio/) was performed to confirm homozygosity of individual *Pif1-*knockout clones.

### Western Blotting and Immunofluorescence.

Western blotting and immunofluorescence staining was performed as described in *SI Appendix*, *Supplemental Materials and Methods*. Primary antibodies used are provided in *SI Appendix*, Table S3. EdU staining was performed with the Click-iT™ EdU Alexa Fluor™ 488 Imaging Kit (Invitrogen, C10337) according to the manufacturer’s protocol.

### Image Analysis with Imaris and FIJI.

Confocal Z-stacks were analyzed using Imaris analysis software. DAPI was used to mask nuclear surfaces and to separate touching surfaces based on the average diameter of nuclei. To quantify the number of abnormal nuclei in images, a machine learning pipeline was created using Imaris’s machine learning filter on DAPI-masked surfaces. ImageJ FIJI software was used to hand-score the number of micronuclei associated with each nucleus and RAD51 foci levels (*SI Appendix*, *Supplemental Materials and Methods*).

### DNA Combing.

Silanized coverslips were produced as described in refs. 68 and 69 with the modification of plasma cleaning coverslips with the Gatan Model 950 Advanced Plasma System with atmospheric air for 10 min. DNA combing was performed as described in *SI Appendix*, *Supplemental Materials and Methods*.

### Cell Viability Assay Using Cell Titer-Glo.

100,000 GCPs per well were grown in opaque-bottom 96-well plates in triplicate and were processed by the addition of 1:1 CellTiter-Glo reagent directly to culture media after 48 to 72 h of treatment. Luminescence values were read on the BioTek Synergy Neo2 microplate reader in the Rockefeller Drug Discovery Resource Center.

### RNA and DNA Sequencing and Analysis.

RNA-sequencing was performed at Genewiz and the Rockefeller University Genomics Resource Center on libraries generated with the NEBNext Ultra II RNA Library Prep Kit for Illumina (NEB #E7770S) from total RNA. RNA-seq analysis was performed at the Rockefeller University Bioinformatics Resource Center using pipelines described in *SI Appendix*, *Supplemental Materials and Methods*. DNA extraction was performed on 4 flash frozen and homogenized mouse medulloblastomas and matched normal forebrain tissue. Illumina WGS library preparation and sequencing was performed at the NIH Intramural Sequencing Center using the Illumina PCR-free TruSeq library preparation kit. Whole-genome sequencing alignment, variant calling, and breakpoint analysis was performed using methods from refs. 70 and 71 and described further in *SI Appendix*, *Supplemental Materials and Methods*.

### Quantification and Statistical Analysis.

ANOVA and *t* tests for statistical significance was performed in Graphpad Prism software. Image quantification was performed as above in FIJI or Imaris and exported to Prism. Significance testing for G4 overlap was performed as above in regioneR (37). Descriptions of statistical analysis presented in the figures are within corresponding figure legends.

## Supplementary Material

Appendix 01 (PDF)

Dataset S01 (XLSX)

Dataset S02 (XLSX)

Dataset S03 (XLSX)

Dataset S04 (XLSX)

## Data Availability

The mouse medulloblastoma RNA-seq dataset has been deposited to GEO under accession GSE288159 (35) and mouse medulloblastoma WGS dataset has been deposited to SRA under accession number PRJNA1214762 (36). Some study data available. HMB1 patient medulloblastoma cell line sequencing data will be shared upon request with proper IRB approval and data sharing agreement.
