# Supplementary material for: G-quadruplexes as a source of vulnerability in BRCA2-deficient granule cell progenitors and medulloblastoma
Source: Proc Natl Acad Sci U S A. 2025 Aug 25;122(35):e2503872122. doi: 10.1073/pnas.2503872122 (PMC12415258; doi:10.1073/pnas.2503872122)
Supplement: Supplementary file 1 — Appendix 01 (PDF) [file pnas.2503872122.sapp.pdf]

PNAS

Supporting Information for

G-quadruplexes as a source of vulnerability in *BRCA2* deficient granule cell progenitors and medulloblastoma

Danielle L. Keahi[1], Mathijs A. Sanders[2, 3], Matthew R. Paul[4], Andrew L. H. Webster[1], Yin Fang[5], Tom F. Wiley[6], Samer Shalaby[7], Thomas S. Carroll[4], Settara C. Chandrasekharappa[8], Carolina Sandoval-Garcia[9], Margaret L. MacMillan[10], John E. Wagner[10], Mary E. Hatten[5#], Agata Smogorzewska[1#]

[1]Laboratory of Genome Maintenance, The Rockefeller University, New York, NY, USA
[2]Cancer, Ageing and Somatic Mutation (CASM), Wellcome Sanger Institute, Hinxton, UK
[3]Department of Hematology, Erasmus MC Cancer Institute, Rotterdam, The Netherlands
[4]Bioinformatics Resource Center, The Rockefeller University, New York, NY, USA
[5]Laboratory of Developmental Neurobiology, The Rockefeller University, New York, NY, USA
[6]Comparative Bioscience Center, The Rockefeller University, New York, NY, USA
[7]Flow Cytometry Resource Center, The Rockefeller University, New York, NY, USA
[8]Cancer Genetics and Comparative Genomics Branch, National Human Genome Research Institute, National Institutes of Health, Bethesda, MD, USA
[9]Department of Neurosurgery, University of Minnesota, Minneapolis, MN, USA
[10]Department of Pediatrics, University of Minnesota, Minneapolis, MN, USA
[#]corresponding author

Correspondence should be addressed to asmogorzewska@rockefeller.edu or hatten@rockefeller.edu

This PDF file includes:

Supporting Materials & Methods
Tables S1 to S3
Figures S1 to S6
Legends for Datasets S1 to S4
SI References

Other supporting materials for this manuscript include the following:

Datasets S1 to S4

Supplemental Materials & Methods

Mouse husbandry and tumor monitoring.

All mouse experiments were approved by the Rockefeller University Institutional Animal Care and Use Committee (IACUC protocol #23039-H). *Brca2*$^{\Delta ex3-4}$ mice (a gift from Dr. Maria Jasin) were crossed with *Trp53*$^{+/-}$ B6/129S mice (B6.129S2-Trp53tm1Tyj/J, Jackson Laboratories) and *hGFAP-Cre* mice (FVB-Tg(GFAP-cre)25Mes/J, Jackson Laboratories) to generate *Brca2*$^{\Delta ex3-4}$ in the CNS coupled with global *Trp53* knockout. Double-knockout animals that form medulloblastomas were checked bi-weekly for cerebellar tumor formation with the IVIS ultrasound system in the Rockefeller University Comparative Bioscience Center. All mice were monitored for weight loss, doming of the head, ataxia, or failure to thrive, and sacrificed humanely. 100 animals were monitored for the survival and tumor outcome cohort. At the humane euthanasia endpoints, tumors were collected and dissociated into primary tumor cell lines, flash frozen for RNA and DNA isolation, or whole brain dissection and fixation for histology. Mice were genotyped using primers in **Table S2**, using GoTaq Green Master Mix (Promega #M7122) for *Brca2, Trp53,* and *hGFAP-Cre* alleles.

Isolation of primary medulloblastoma cells.

Primary medulloblastoma tumor cells were isolated as previously described in (1). Briefly, after mice were humanely sacrificed, friable tumors were dissected from the cerebellum and dissociated with Accutase for 5min at 37C. Tumors were dissociated in Accutase with pipetting and were spun down for 5min at 500g. Tumor cells were plated on tissue-culture 6-well plates pre-coated in 10ug/mL laminin (Sigma L2020) in NeuroCult Mouse/Rat Proliferation Kit medium (STEMCELL Technologies #05702) with 1x Pen/Strep, epidermal growth factor (EGF, Peprotech #AF-100-15), basic fibroblast growth factor, recombinant human (bFGF, Peprotech #100-18B), and heparin (STEMCELL Technologies, #07980).

FA-D1 Patient Medulloblastoma Primary Cells

FA-D1 patient F89P1 was entered into the Rockefeller University International Fanconi Anemia Registry (IFAR) under IRB number AAU-0112. Medulloblastoma tissue was collected after surgical resection and primary cells were isolated in the same manner as for mouse MBs as described below. Primary human MB cells (HMB1) were cultured in NeuroCult NS-A Proliferation Kit (Human, STEMCELL Technologies #NC0668437) with 1x Pen/Strep, epidermal growth factor (EGF, Peprotech #AF-100-15), basic fibroblast growth factor, recombinant human (bFGF, Peprotech #100-18B), and heparin (STEMCELL Technologies, #07980).

Isolation of primary GCPs.

GCPs were isolated at P7 from C57BL/6 (strain #000664, Jackson Laboratories) wild-type or *hGFAP-Cre; Brca2*$^{\Delta ex3-4}$*; Trp53* mutant animals as previously described (2). Cerebella were dissected in ice-cold CMF-PBS and dissociated with trypsin-DNAse I for 5min at 37C. Dissociated cerebella were centrifuged for 5min at 700g at 4C. Trituration of cerebellar tissue was performed in CMF-PBS with DNase I with fine fire-polished Pasteur pipets followed by extra-fine fire-polished Pasteur pipets. GCPs were enriched using the Percoll separation gradient. The GCP-containing small cell fraction was collected and pre-plated on a Petri dish for 30min followed by one hour on a tissue culture-treated dish, allowing for plating of fibroblasts and other contaminating cells. After preplating, GCPs were pelleted at 700g for 5min at 4C and counted prior to culturing.

Culture of GCPs on coverslips.

GCPs were cultured on glass coverslips pre-coated with poly-D lysine (0.1mg/mL, Sigma #P1024) for 1h followed by drying and Matrigel coating (growth factor-reduced, Corning #354230). GCPs were cultured in Basal Medium Eagle (Gibco #21010-046) with 2mM L-

glutamine (Gibco #25030-016), 1X Pen-Strep (Gibco #15140-015), 0.9% glucose (Sigma #G8769), 10% horse serum (Gibco #16050-122, heat-inactivated) and 5% fetal bovine serum (Gibco #26140-079, heat-inactivated)) at a final concentration of 50,000 cells per coverslip in 500ul media. GCPs were treated with 100nM SAG (Cayman Chemical, #11914) for 3h or 12h as well as pyridostatin (MedChemExpress #HY-15176A) at 2.5uM, 5uM, or 10uM. EdU (ThermoFisher #E10187) was added at 10uM 30min prior to the end of each timepoint.

Culture of re-aggregate GCPs in suspension.
GCPs were plated at $2*10^6$ cells in 500ul of media in Ultra Low Attachment 24-well plates (Corning #3473) or 100,000 cells in 100ul in opaque-bottom 96-well plates (ThermoFisher #15042) and allowed to re-aggregate for 16h prior to treatment with 100nM SAG, mitomycin C, cisplatin, or pyridostatin. Re-aggregate GCPs grown in 24-well plates were pulsed with 10uM EdU at the end of the treatment window and fixed in 4% methanol-free PFA for 15min prior to being centrifuged onto slides with the ThermoFisher Cytospin 4 Cytocentrifuge at the Rockefeller University Flow Cytometry Resource Center. Re-aggregate GCPs grown in 96-well plates were processed for cell viability readings in CellTiter-Glo as described below.

Generation of Pif1-KO MB Cells.
CRISPR RNP cutting was performed with Cas9 complexed with an assembled sgRNA using the IDT Pre-Designed Alt-R CRISPR/Cas9 system. mPif1 guides “AC” and “AA” were selected from the gRNA design tool due to high specificity and predicted cutting efficiency (Mm.Cas9.PIF1.1.AA - “sgPif1-N” and Mm.Cas9.PIF1.1.AC - “sgPif1-H”). gRNAs were complexed in oligo duplex buffer (IDT) with a tdTomato-labeled tracrRNA (IDT #1075927) prior to complexing with SpCas9 V3 (IDT #1081059). CRISPR-RNP complexes were delivered to primary medulloblastoma cells using the P3 Primary Cell Nucleofection Kit (Lonza #V4XP-3032) for the 4D Nucleofector X (Lonza #AAF-1003X). Program optimization was performed with pMaxGFP plasmid included in the nucleofection kit and the program CL-133 was found to be the optimized protocol for primary MB cells. CRISPR cutting efficiency was determined with Synthego ICE analysis using 700bp of sequenced PCR around the cut site for each guide.

Western blotting.
Protein lysates were generated by sonicating and boiling 1*10^6 cells in 100ul of 2X Laemmli Buffer (Bio-Rad) with BME. 20ul of lysate was added to the wells of precast 10-well 4-12% Bis-Tris PAGE gels in MOPS running buffer. After overnight transfer, membranes were blocked in 5% milk in TBST (10mM Tris-Hcl pH 7.5, 150mM NaCl, 0.1% Tween-20) and incubated in primary antibodies overnight at 4C with rocking (**Supplemental Table 7**). Membranes were washed in TBST 3x5min before being incubated with HRP-conjugated secondary antibodies for 1h at RT with rocking. Membranes were washed again in TBST and detected by enhanced chemiluminescence. Western blots were visualized with the Azure c300 imaging system.

Immunofluorescence.
Coverslips were fixed in 4% paraformaldehyde in PBS for 15min at RT. Permeabilization and block were performed in 3% BSA in PBS with 0.5% Triton-X for 30min at RT with rocking. Coverslips were then incubated with primary antibodies diluted in 3% BSA overnight at 4C (**Supplemental Table 7**). Washes were performed in PBS 3x5min. Secondary antibodies were diluted in 3% BSA in PBS and incubated for 1h at RT and washes were performed 3x5min in PBS. Hoechst 33342 (ThermoFisher) was used to stain DNA and coverslips were incubated in 1:2000 diluted Hoechst in PBS for 20min at RT at the end of the wash. Coverslips were mounted on SuperFrost Plus slides in Fluoromount-G and allowed to cure overnight. Imaging was performed on an LSM 880 confocal microscope at the Rockefeller University Bio-imaging Resource Center.

EdU Staining.
Cells pulsed with 10uM EdU for 30min were washed in 1x PBS and fixed in 3.7% formaldehyde in PBS for 15min at RT. Cells were stained with Click-iT™ EdU Alexa Fluor™ 488 Imaging Kit

(Invitrogen, C10337) according to manufacturer's protocol. DNA was stained with Hoechst 33342 and coverslips were mounted in Fluoromount-G.

Image analysis with Imaris.
Confocal Z-stacks were analyzed using Imaris imaging software. DAPI was used to mask nuclear surfaces and to separate touching surfaces based on the average diameter of nuclei. To quantify the number of abnormal nuclei in images, a machine learning pipeline was created using Imaris's machine learning filter on DAPI-masked surfaces. 200 normal and 200 abnormal nuclei were selected to train the machine learning pipeline and quantification of the trained pipeline was validated in a subset of training samples with hand-scoring. On subsequent datasets, the machine learning filter was run in an automated way to reduce bias in image quantification. Within each nuclear surface, mean intensity values for the other immunofluorescence channels such as yH2AX and EdU were also automatically quantified and exported.

Image Analysis in FIJI.
Micronuclei were hand-scored in FIJI by first counting the total number of nuclei (at least 300 nuclei per three biological replicates) with the CellCounter plugin and then counting cells with at least one micronucleus associated in the immediate vicinity of the nucleus. Micronuclei were reported as percent of cells containing one micronucleus. Due to the low cytoplasmic volume of MB primary tumor cells, micronuclei were clearly associated with the nucleus. RAD51 foci were additionally hand-scored, with total nuclei counted with the CellCounter plugin followed by counting of cells with greater than 5 bright RAD51-positive foci. 100 cells were scored per three biological replicates for this assay.

DNA combing coverslip production.
Silanized coverslips were produced as described in (3, 4) with the modification of plasma cleaning coverslips with the Gatan Model 950 Advanced Plasma System with atmospheric air for 10 minutes.

DNA combing.
Cells were labeled with IdU (100uM) and CldU (100uM) for the specified pulse times while in exponential growth. Cells were harvested in Accutase and washed in PBS prior to resuspension in 45ul combing Resuspension Buffer (PBS with 0.2% sodium azide). 45ul of 2% low melt agarose was added to briefly warmed cell suspension and poured into agarose plug molds. Agarose plugs were digested overnight at 55°C in 1mg/mL proteinase K, 1% N-Lauroylsarcosine, 0.2% sodium deoxycholate, 100mM EDTA, 10mM Tris-HCl, pH 7.5. Plugs were wash three times for at least an hour in 1X TE pH 8.5 with 100mM NaCl. Plug melting was performed in 1mL freshly prepared combing buffer (0.5M MES pH 5.5) for 68°C for 20 minutes followed by overnight incubation with 1ul beta-agarase at 42°C. Combing was performed onto silanized coverslips made in house using the Molecular Combing System (Genomic Vision). Slides were dried for 2h at 65°C and denatured in 0.5 M NaOH + 1M NaCl for 8min or moved directly into YOYO-1 DNA stain (ThermoFisher) for quality control check. Denatured combed coverslips were dehydrated in 70%, 90%, and 100% ethanol for 5min sequentially and dried in the dark at RT. Blocking was performed at RT for 1h in 5% FBS in PBS and primary antibodies were incubated overnight diluted in 5% FBS in PBS. Coverslips were washed 3x5min in 5% FBS in PBS prior to 1h secondary antibody incubation at RT. Coverslips were mounted in Fluoromount-G and imaged on an Inverted Olympus IX-71 DeltaVision (Applied Precision) microscope. Combed DNA fibers were scored in FIJI software.

Cell viability assay using Cell Titer-Glo.
100,000 GCPs per well were grown in opaque-bottom 96-well plates in triplicate and were processed by the addition of 1:1 CellTiter-Glo reagent directly to culture medium after 48-72 hours of treatment. Plates were agitated for 30min at RT to allow cell lysis to occur prior to reading luminescence values on the BioTek Synergy Neo2 microplate reader in the Rockefeller Drug Discovery Resource Center.

RNA-sequencing.
Total RNA was isolated using the Zymo Quick-RNA Miniprep Kit with on-column DNA digestion. mRNA was enriched with the NEBNext Poly(A) mRNA Magnetic Isolation Module (NEB #E7490) and library preparation was performed with the NEBNext Ultra II RNA Library Prep Kit for Illumina (NEB #E7770S). Indices used were the NEBNext Multiplex Oligos for Illumina (NEB #E7335, #E7500). Sequencing libraries were evaluated on the Agilent 2200 TapeStation with D1000 High Sensitivity ScreenTape and sequenced on a NextSeq 500 High Output sequencer to generate 75bp reads at the Rockefeller University Genomics Resource Center. Transcript abundance was determined using Salmon (v0.8.1) and the reference transcript sequences from TxDb.Mmusculus.UCSC.mm10.knownGene (v3.4.0) (5). Transcript counts from Salmon were imported into R with the tximport R Bioconductor package (v1.20), and differentially expressed genes were determined with the DESeq2 R Bioconductor package (v1.20) (6). Significant genes were considered as p-adj < 0.01 and log2FC ≥ 1. GO gene set enrichment analysis (GSEA) was performed in clusterProfiler (v4.0.5) (7). Heatmaps were generated in pheatmap (v1.0.12) (8).

Illumina whole-genome sequencing.
DNA extraction was performed on 4 flash frozen mouse medulloblastomas and matched forebrain normal controls using UltraPure phenol:chloroform:isoamyl alcohol (25:24:1) (ThermoFisher 15593031). Frozen tumor tissue was homogenized with bead-beating using the Qiagen TissueLyser II in Qiagen DNeasy Blood and Tissue Kit Buffer ATL with proteinase K added. Illumina WGS library preparation and sequencing was performed at the National Institutes of Health (NIH) Intramural Sequencing Center using the Illumina PCR-free TruSeq library preparation kit. Tumor samples were sequenced to 60x coverage of the genome and matched normal samples were sequenced to 30x genome coverage.

Whole-genome sequencing alignment and mutation calling
Sequencing data from mouse was aligned to NCBI mouse reference genome GRCm38 (mm10) and sequencing data from the HMB1 primary human MB line was performed using GRCh37 reference genome (hg19) using BWA-MEM2 (https://github.com/bwa-mem2/bwa-mem2). Duplicate reads were identified with (https://github.com/samtools/samtools). Somatic SNVs were called with CaVEMan (https://github.com/cancerit/CaVEMan) and somatic indels called by Pindel (https://github.com/cancerit/cgpPindel). SVs were called with BRASS (https://github.com/cancerit/BRASS) and further annotated by AnnotateBRASS (https://github.com/MathijsSanders/AnnotateBRASS) as described previously (9). After variant calling, filtering of artefacts, alignment errors, and low-quality variants was done as described previously (9, 10) followed by filtering of sample-matched normal forebrain controls for mouse samples. For the human MB primary cell line HMB1, which lacked a paired normal control, variant filtering was performed with an in-house human panel of controls (10). CNV regions were determined with CNVkit (11).

Whole-genome sequencing breakpoint analysis and G4 overlap
Mutation breakpoints were determined using the start and end sites of SVs and from start sites of small indels. CNV regions were excluded from breakpoint analysis because breakpoint ends are not exactly determined in CNVkit but estimated from region coverage differences. Each mutation endpoint was adjusted to a region of 100bp or 1Kb centered on the endpoint in GenomicRanges (12). Experimental sequenced LiK and PDS G4 datasets were accessed from (13) and computation PQS G4s were determined using sequences derived from the BSgenome.Hsapiens.UCSC.hg19 (v1.4.3) and Bsgenome.Mmusculus.UCSC.mm10 (v.1.4.0) packages and entered into pqsfinder (v2.4.0) (14) with minimum score of 20 used to identify all G4s in a given sequence region. Overlap analysis was performed in GenomicRanges. Visualizations were performed in GViz (v1.36.2) (15). Overlap significance testing was performed using the regioneR package (v1.2.4) (16), to do permutation testing with 1000 iterations of random shuffling.

Quantification and statistical analysis

ANOVA and t-tests for statistical significance was performed in Graphpad Prism software. Image quantification was performed as above in FIJI or Imaris and exported to Prism. Significance testing for G4 overlap was performed as above in regioneR (16). Descriptions of statistical analysis presented in the figures are within corresponding figure legends.

Table S1. sgPif1 cutting efficiency.

Homozygous clone	Guide RNA	Mutation location	Stop codon position (aa)
N2	sgNeg	N/A	N/A
N4	sgNeg	N/A	N/A
N14	sgNeg	N/A	N/A
C1	sgPif1-H	-7	357
C3	sgPif1-H	-7	357
C8	sgPif1-H	-11	322
C10	sgPif1-H	-4	358
**Bulk CRISPR**	**Cutting efficiency**	**Knockout score**	**Most abundant indel**
sgNeg	N/A	N/A	N/A
sgPif1-H	95%	85%	+1

Table S2: Primers

Name	Sequence	Vendor
Cre genotyping F	GGACATGTTCAGGGATCGCCAGGCG	IDT
Cre genotyping R	GCCAGATTACGTATATCCTGGCAGCG	IDT
BRCA2 genotyping 4F	TGTTGCTAGTCCGCCTCTGAA	IDT
BRCA2 genotyping 4R	GCCTGGGCTGGCTTCATACT	IDT
P53 genotyping mut F	CAGCCTCTGTTCCACATACACT	IDT
P53 genotyping WT F	AGGCTTAGAGGTGCAAGCTG	IDT
P53 genotyping R	TGGATGGTGGTATACTCAGAGC	IDT
mPif1 Gateway F primer	GGGGACAAGTTTGTACAAAAAAGCAGGC TTCACC**ATGCGCTCCGGTCTCTGCACG**	IDT
mPif1 Gateway R primer	GGGGACCACTTTGTACAAGAAAGCTGGGTC GAGGTTTGGGTCCATGTTCTC	IDT
mPif1 E316Q mutagenesis primer F	ctccaccatggagatctggtcaatgaccaaacgtt	IDT
mPif1 E316Q mutagenesis primer R	aacgtttggtcattgaccagatctccatggtggag	IDT
mPif1 genotyping primer F for sgPif1-H	GGGGAGCAGGCAGGCAACT	IDT
mPif1 genotyping primer R for sgPif1-H	TGGTCACTGGTGGCAACTGTAGGA	IDT
mPif1 genotyping primer F for sgPif1-N	GACGTTTCTCCTCGCTCCAG	IDT
mPif1 genotyping primer R for sgPif1-N	CAGCGTGCGCAGGAAGC	IDT

Table S3: Antibodies

Name	Vendor	Catalog #
Mouse monoclonal anti-yH2AX Ser139 (clone JBW301), IF 1:2000	Millipore Sigma	05-636
Rat monoclonal anti-BrdU [BU1/75 (ICR1)], combing: 1:20	Abcam	ab6326
Mouse monoclonal anti-BrdU (B44), combing: 1:10	BD Biosciences	347580
Goat Anti-Mouse IgG H&L Cross-Absorbed (Alexa Fluor® 488), IF:1:1000, combing:1:100	ThermoFisher	A-11029
Goat Anti-Rat IgG H&L Cross-Absorbed (Alexa Fluor® 594), IF: 1:1000, combing: 1:100	ThermoFisher	2107787
SYBR Gold (1:10,000)	ThermoFisher	2107787
Hoechst 33342 (1:2,000)	ThermoFisher (Click-it EdU kit)	C10337

Supporting Figures

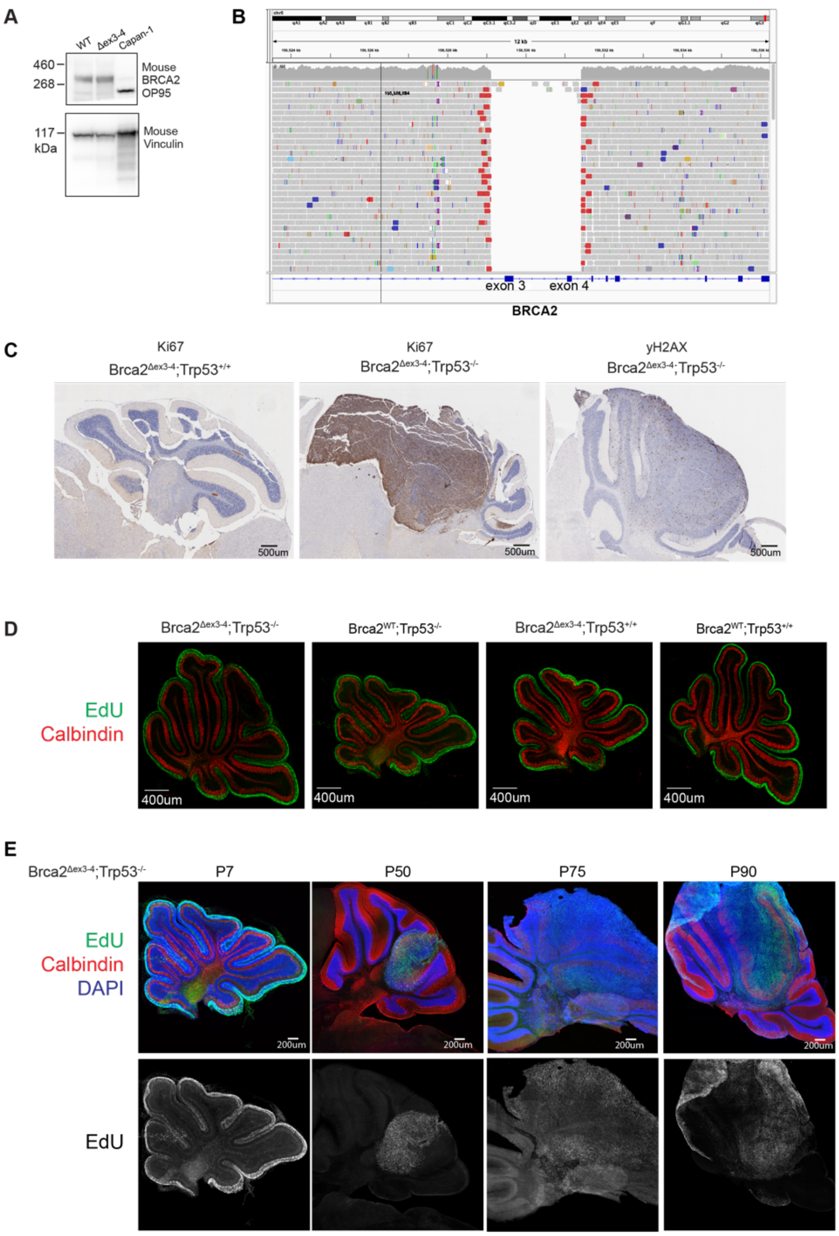
A
WT
Δex3-4
Capan-1
460
268
Mouse BRCA2 OP95
117
kDa
Mouse Vinculin
B
exon 3
exon 4
BRCA2
C
Ki67
Brca2Δex3-4;Trp53+/+
Ki67
Brca2Δex3-4;Trp53-/-
yH2AX
Brca2Δex3-4;Trp53-/-
500um
D
Brca2Δex3-4;Trp53-/-
Brca2WT;Trp53-/-
Brca2Δex3-4;Trp53+/+
Brca2WT;Trp53+/+
EdU
Calbindin
400um
E
Brca2Δex3-4;Trp53-/-
P7
P50
P75
P90
EdU
Calbindin
DAPI
200um
EdU

Fig. S1: *Brca2* mutation leads to mild cerebellar hypoplasia and tumor development when crossed with *Trp53-/-*

A: Western blot of cerebellar lysates from *Brca2*^{WT}; *Trp53*$^{+/+}$ and *Brca2*$^{\Delta ex3\text{-}4}$; *Trp53*$^{+/+}$ mice at postnatal day 8 (P8) and control CAPAN-1 *BRCA2* truncation mutant cell line lysate. *Brca2*$^{\Delta ex3\text{-}4}$ expresses a similar sized BRCA2 peptide as *Brca2*^{WT}.

B: IGV display of the deep deletion of BRCA2 exon 3 and 4 mediated by hGFAP-Cre recombination of loxP sites in a representative *Brca2*$^{\Delta ex3\text{-}4}$; *Trp53*$^{-/-}$ tumor WGS sample.

C: Ki67 and yH2AX histology demonstrates positive staining within the tumor in double-mutant animals. Sections were collected from three separate animals at P90. The left section from *Brca2*$^{\Delta ex3\text{-}4}$; *Trp53*$^{+/+}$ animal originates from the brain also shown in the left panel of Figure 1F, and the middle section from *Brca2Δ*$^{ex3\text{-}4}$; *Trp53*$^{-/-}$ originates from the brain also shown in the right panel of Figure 1F.

D: EdU-pulsed P7 cerebellar sagittal sections of mice from different genotypes shows mild hypoplasia in mice with the *Brca2*$^{\Delta ex3\text{-}4}$; *Trp53*$^{+/+}$ genotype. EdU is in green and calbindin (Purkinje neuron marker) is in red.

E: EdU timecourse assay in *Brca2*$^{\Delta ex3\text{-}4}$; *Trp53*$^{-/-}$ animals at P7, P50, P75, P90 highlights proliferative cells over time with GCPs marked by EdU at P7 and transformed GCPs at P50, P75, and P90. EdU is in green, calbindin in red, and DAPI in blue. At the P50 timepoint, a small tumor mass can be observed as inappropriately EdU-positive in the otherwise post-mitotic cerebellum. This tumor mass grows aggressively into the surrounding cerebellar tissue before mice exhibit symptoms of sickness between P75 and P90 and are humanely sacrificed.

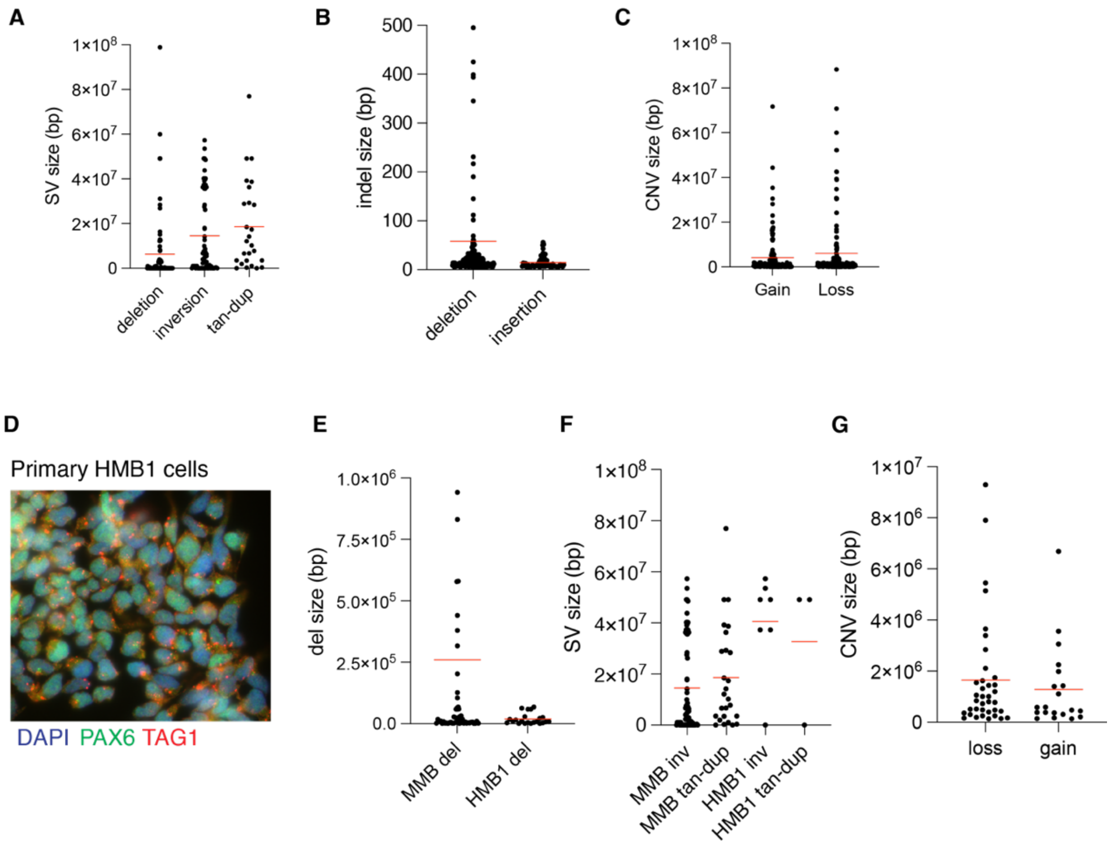
A
SV size (bp)
1×10^8
8×10^7
6×10^7
4×10^7
2×10^7
0
deletion
inversion
tan-dup
B
indel size (bp)
500
400
300
200
100
0
deletion
insertion
C
CNV size (bp)
Gain
Loss
D
Primary HMB1 cells
DAPI PAX6 TAG1
E
del size (bp)
1.0×10^6
7.5×10^5
5.0×10^5
2.5×10^5
0.0
MMB del
HMB1 del
F
SV size (bp)
MMB inv
MMB tan-dup
HMB1 inv
HMB1 tan-dup
G
CNV size (bp)
1×10^7
8×10^6
6×10^6
4×10^6
2×10^6
0
loss
gain

Fig. S2. Mouse and human medulloblastoma mutation analysis
A: Indel sizes from MMB1-MMB4.
B: Structural variant sizes from four mouse medulloblastomas (MMB1-4).
C: Copy number variation region sizes from MMB1-MMB4.
D: Immunofluorescence image of primary human MB cells (HMB1) isolated from a FA-D1 patient demonstrates positive staining with the GCP markers PAX6 and TAG1.
E: SV deletion size comparison of collected MMB1-4 deletion breakpoints and HMB1 deletions.
F: SV inversion and tandem-duplication size comparisons between MMB1-4 and HMB1.
G: CNV region sizes from HMB1.

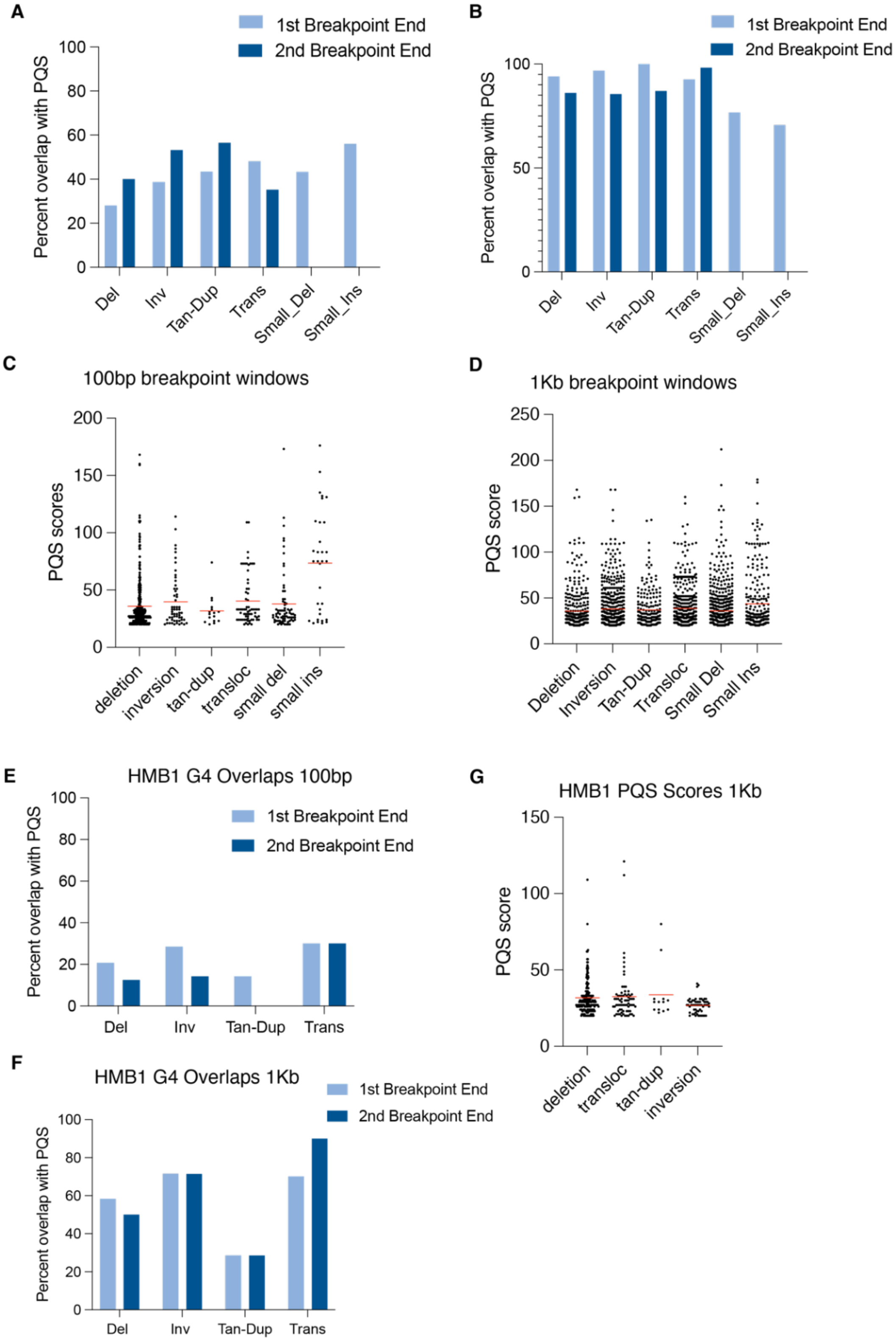
A
1st Breakpoint End
2nd Breakpoint End
Percent overlap with PQS
0
20
40
60
80
100
Del
Inv
Tan-Dup
Trans
Small_Del
Small_Ins
B
1st Breakpoint End
2nd Breakpoint End
Percent overlap with PQS
0
50
100
Del
Inv
Tan-Dup
Trans
Small_Del
Small_Ins
C
100bp breakpoint windows
PQS scores
0
50
100
150
200
deletion
inversion
tan-dup
transloc
small del
small ins
D
1Kb breakpoint windows
PQS score
0
50
100
150
200
250
Deletion
Inversion
Tan-Dup
Transloc
Small Del
Small Ins
E
HMB1 G4 Overlaps 100bp
1st Breakpoint End
2nd Breakpoint End
Percent overlap with PQS
0
20
40
60
80
100
Del
Inv
Tan-Dup
Trans
G
HMB1 PQS Scores 1Kb
PQS score
0
50
100
150
deletion
transloc
tan-dup
inversion
F
HMB1 G4 Overlaps 1Kb
1st Breakpoint End
2nd Breakpoint End
Percent overlap with PQS
0
20
40
60
80
100
Del
Inv
Tan-Dup
Trans

Fig. S3. Mouse and human medulloblastoma G4 overlap

A: Percent overlap of PQS G4s and 100bp breakpoint windows of each breakpoint end per mutation type of SV and per single breakpoint end for indels (small_del and small_ins).

B: Percent overlap of PQS G4s and 1Kb breakpoint windows of each breakpoint end per mutation type of SV and per single breakpoint end for indels (small_del and small_ins).

C: Individual PQS scores for collected 100bp breakpoint windows of each mutation type.

D: Individual PQS scores for collected 1Kb breakpoint windows of each mutation type.

E: Percentage PQS G4 overlap for each end of breakpoints per mutation type for 100bp windows in HMB1.

F: Percentage PQS G4 overlap for each end of HMB1 breakpoints per mutation type for 1 Kb windows.

G: All PQS scores within 1Kb breakpoint windows per mutation type demonstrates a similar distribution of PQS stability score for each mutation type.

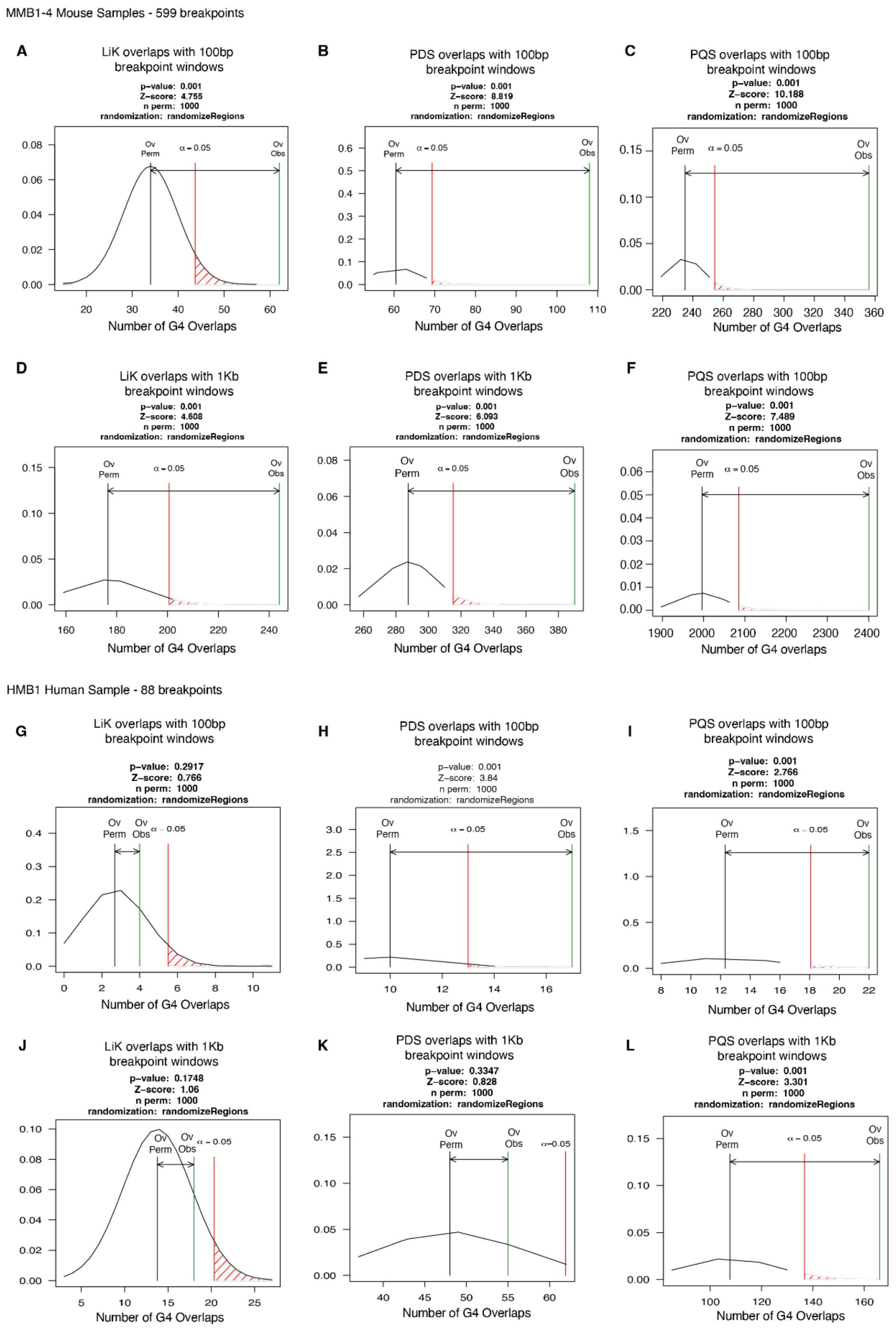
MMB1-4 Mouse Samples - 599 breakpoints
A
LiK overlaps with 100bp breakpoint windows
p–value: 0.001
Z–score: 4.755
n perm: 1000
randomization: randomizeRegions
Number of G4 Overlaps
B
PDS overlaps with 100bp breakpoint windows
p–value: 0.001
Z–score: 8.819
n perm: 1000
randomization: randomizeRegions
Number of G4 Overlaps
C
PQS overlaps with 100bp breakpoint windows
p–value: 0.001
Z–score: 10.188
n perm: 1000
randomization: randomizeRegions
Number of G4 Overlaps
D
LiK overlaps with 1Kb breakpoint windows
p–value: 0.001
Z–score: 4.608
n perm: 1000
randomization: randomizeRegions
Number of G4 Overlaps
E
PDS overlaps with 1Kb breakpoint windows
p–value: 0.001
Z–score: 6.093
n perm: 1000
randomization: randomizeRegions
Number of G4 Overlaps
F
PQS overlaps with 100bp breakpoint windows
p–value: 0.001
Z–score: 7.489
n perm: 1000
randomization: randomizeRegions
Number of G4 overlaps
HMB1 Human Sample - 88 breakpoints
G
LiK overlaps with 100bp breakpoint windows
p–value: 0.2917
Z–score: 0.766
n perm: 1000
randomization: randomizeRegions
Number of G4 Overlaps
H
PDS overlaps with 100bp breakpoint windows
p–value: 0.001
Z–score: 3.84
n perm: 1000
randomization: randomizeRegions
Number of G4 Overlaps
I
PQS overlaps with 100bp breakpoint windows
p–value: 0.001
Z–score: 2.766
n perm: 1000
randomization: randomizeRegions
Number of G4 Overlaps
J
LiK overlaps with 1Kb breakpoint windows
p–value: 0.1748
Z–score: 1.06
n perm: 1000
randomization: randomizeRegions
Number of G4 Overlaps
K
PDS overlaps with 1Kb breakpoint windows
p–value: 0.3347
Z–score: 0.828
n perm: 1000
randomization: randomizeRegions
Number of G4 Overlaps
L
PQS overlaps with 1Kb breakpoint windows
p–value: 0.001
Z–score: 3.301
n perm: 1000
randomization: randomizeRegions
Number of G4 Overlaps

Fig. S4: Statistical testing of WGS breakpoint overlap with G4s
A: regioneR statistical test for overlap of experimental LiK-stabilized G4s and 100bp breakpoints from MMB1 SVs and indels tested shows significant overlap of G4s and breakpoints over random shuffling.
B: regioneR statistical test for overlap of experimental PDS-stabilized G4s and 100bp breakpoints from MMB1 SVs and indels tested over random shuffling of breakpoint windows.
C: regioneR statistical test for overlap of pqsfinder identified G4s and 100bp breakpoints from MMB1 SVs and indels tested over random shuffling of breakpoint windows.
D: regioneR statistical test for overlap of experimental LiK-stabilized G4s and 1Kb breakpoints from MMB1 SVs and indels tested over random shuffling of breakpoint windows.
E: regioneR statistical test for overlap of experimental PDS-stabilized G4s and 1Kb breakpoints from MMB1 SVs and indels tested over random shuffling of breakpoint windows.
F: regioneR statistical test for overlap of pqsfinder identified G4s and 1Kbbreakpoints from MMB1 SVs and indels tested over random shuffling of breakpoint windows.
G: regioneR statistical test for overlap of experimental LiK-stabilized G4s and 100bp breakpoints from HMB1 SVs tested over random shuffling of breakpoint windows. Non-significant p-value.
H: regioneR statistical test for overlap of experimental PDS-stabilized G4s and 100bp breakpoints from HMB1 SVs tested over random shuffling of breakpoint windows.
I: regioneR statistical test for overlap of pqsfinder identified G4s and 100bp breakpoints from MMB1 SVs and indels tested over random shuffling of breakpoint windows.
J: regioneR statistical test for overlap of experimental LiK-stabilized G4s and 1Kb breakpoints from HMB1 SVs tested over random shuffling of breakpoint windows. Non-significant p-value.
K: regioneR statistical test for overlap of experimental PDS-stabilized G4s and 1Kb breakpoints from HMB1 SVs tested over random shuffling of breakpoint windows. Non-significant p-value.
L: regioneR statistical test for overlap of pqsfinder identified G4s and 1Kb breakpoints from HMB1 SVs tested over random shuffling of breakpoint windows.

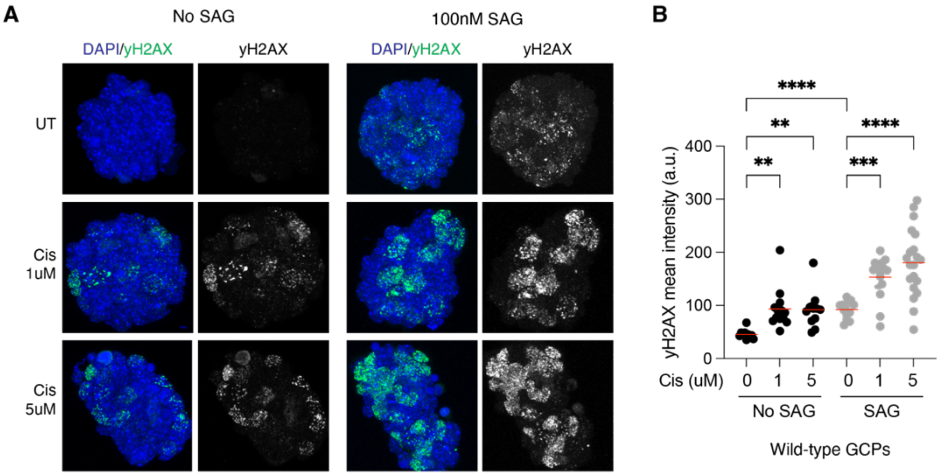
A
No SAG
100nM SAG
DAPI/yH2AX
yH2AX
DAPI/yH2AX
yH2AX
UT
Cis 1uM
Cis 5uM
B
****
**
****
**
***
yH2AX mean intensity (a.u.)
400
300
200
100
0
Cis (uM)
0
1
5
0
1
5
No SAG
SAG
Wild-type GCPs

Fig. S5: GCP re-aggregates demonstrate increased yH2AX under SAG treatment.
A: Confocal image at 63x magnification of EdU-pulsed wild-type re-aggregates treated with SAG and increasing concentrations of cisplatin show an increase in yH2AX intensity in cells treated with SAG. EdU is in green and DAPI is in blue.
B: Quantification of imaging demonstrates a significant increase in yH2AX mean intensity in SAG-treated GCP re-aggregates.

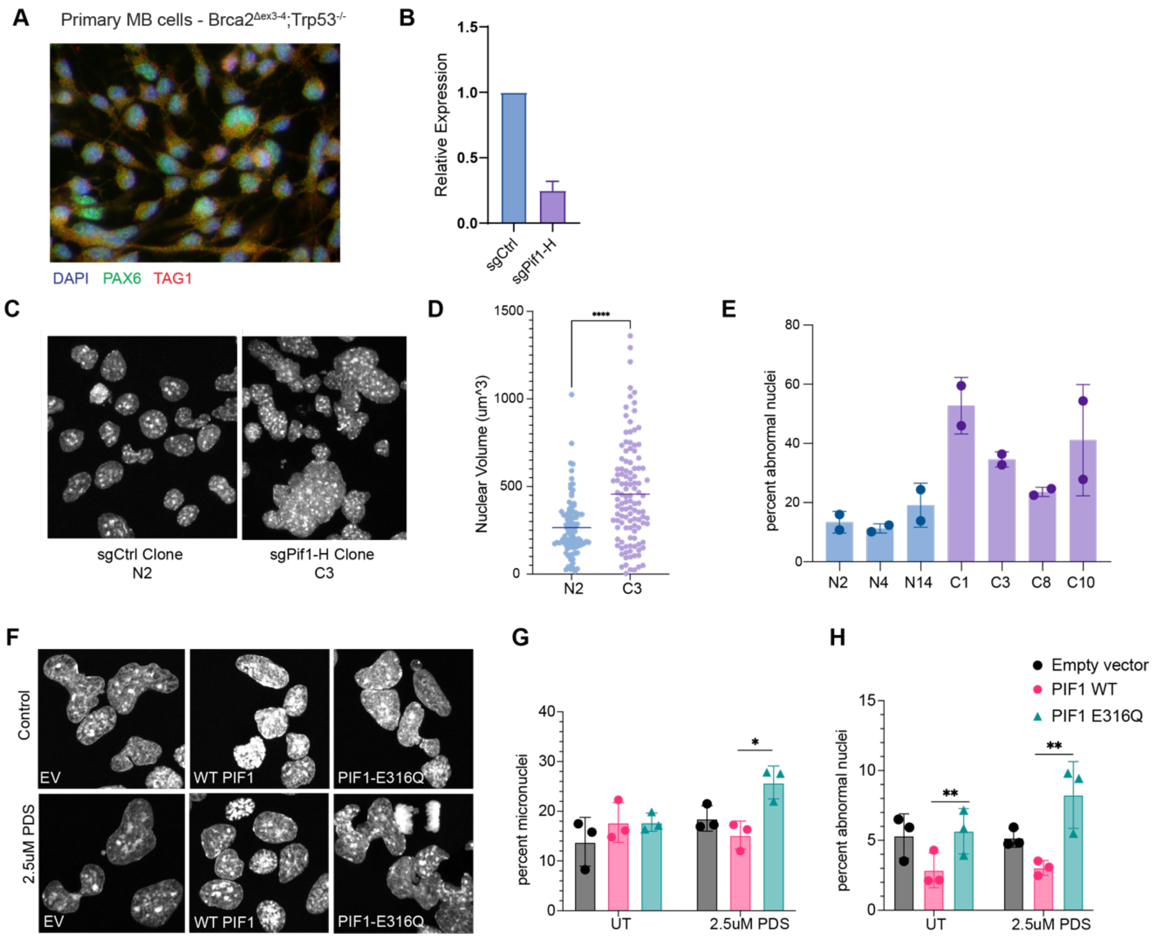

A
Primary MB cells - Brca2Δex3-4;Trp53-/-
DAPI PAX6 TAG1
B
Relative Expression
1.5
1.0
0.5
0.0
sgCtrl
sgPif1-H
C
sgCtrl Clone N2
sgPif1-H Clone C3
D
Nuclear Volume (um^3)
1500
1000
500
0
****
N2
C3
E
percent abnormal nuclei
80
60
40
20
0
N2 N4 N14 C1 C3 C8 C10
F
Control
2.5uM PDS
EV
WT PIF1
PIF1-E316Q
G
percent micronuclei
40
30
20
10
0
UT
2.5uM PDS
H
Empty vector
PIF1 WT
PIF1 E316Q
percent abnormal nuclei
15
10
5
0
UT
2.5uM PDS

Fig. S6:
A: Immunofluorescence image of primary MB cells stained with the GCP markers PAX6 and TAG1 demonstrates positive staining of neuronal progenitor-like MB cells.
B: Relative expression of Pif1 transcript as determined by qPCR of PIF1 sgCtrl clone N2 and sgPif1-H clone C3.
C: Representative 63x confocal images of DAPI-stained nuclei from sgNeg clone N2 and sgPif1-H clone C3. Large abnormal nuclei and micronuclei can be observed exclusively in the C3 clone, representative of 4 sgPif1-H clones analyzed. Three negative control clones were analyzed and were found to lack an increase in abnormal nuclei.
D: Nuclear volume measured in Imaris on Z-stacks of 63x images captured on the Zeiss LSM 880 confocal microscope from DAPI-stained nuclei demonstrates a significant increase in nuclear volume in the sgPif1-H clone C3.
E: The percentage of abnormal nuclei measured using the abnormal nuclei machine learning filtering pipeline in Imaris. There is an increase in abnormal nuclei in sgPif1-H derived clones (C1, C3, C8, C10) and a low baseline level of abnormal nuclei in sgNeg (N2, N4, N14) clones.
F: 63x IF images of representative sgPif1-H clone C1 cells stained with DAPI after 48h of treatment with and without 2.5uM PDS. Cells complemented with empty vector or PIF1-E316Q demonstrate genomic instability phenotypes.
G: Micronuclei levels in sgPif1-H clones (C1 shown as a representative clone) remain high after complementation with PIF1-WT and increase in PIF1-E316Q complemented cells after 48h of treatment with 2.5uM PDS.
H: Abnormal nuclei percentage decreases only after complementation with PIF1-WT and increases after PDS treatment with PIF1-E316Q complementation.

Dataset S1 (separate file): Medulloblastoma Subgroup Gene Clustering
Medulloblastoma subgroup genes in tumor and control samples. Attached as Excel file of gene IDs, TPMs, and cluster number.

Dataset S2 (separate file): GCP Developmental Gene Clustering
GCP developmental genes in tumor and developing GCPs. Attached as Excel file of gene IDs, TPMs, and cluster number.

Dataset S3 (separate file): Mouse mutations (MMB1-4)
Attached Excel file of structural variants, copy number variation, and indels from 4 mouse tumors with matched normal forebrain filtering. G4 overlaps over the full mutations and at mutation breakends are also reported.

Dataset S4 (separate file): Human MB mutations (HMB1)
Attached Excel file of structural variants and copy number variation reported from 1 human MB primary cell line with no matched normal. G4 overlaps over the full mutations and at mutation breakends are also reported.

SI References

1. S. Badodi, S. Marino, L. Guglielmi, Establishment and Culture of Patient-Derived Primary Medulloblastoma Cell Lines. *Methods Mol Biol* **1869**, 23-36 (2019).
2. M. E. Hatten, Neuronal regulation of astroglial morphology and proliferation in vitro. *J Cell Biol* **100**, 384-396 (1985).
3. G. Moore, J. Jimenez Sainz, R. B. Jensen, DNA fiber combing protocol using in-house reagents and coverslips to analyze replication fork dynamics in mammalian cells. *STAR Protoc* **3**, 101371 (2022).
4. D. Gallo, G. Wang, C. M. Yip, G. W. Brown, Analysis of Replicating Yeast Chromosomes by DNA Combing. *Cold Spring Harb Protoc* **2016**, pdb prot085118 (2016).
5. R. Patro, G. Duggal, M. I. Love, R. A. Irizarry, C. Kingsford, Salmon provides fast and bias-aware quantification of transcript expression. *Nat Methods* **14**, 417-419 (2017).
6. M. I. Love, W. Huber, S. Anders, Moderated estimation of fold change and dispersion for RNA-seq data with DESeq2. *Genome Biol* **15**, 550 (2014).
7. G. Yu, L. G. Wang, Y. Han, Q. Y. He, clusterProfiler: an R package for comparing biological themes among gene clusters. *OMICS* **16**, 284-287 (2012).
8. R. Kolde, pheatmap: Pretty Heatmaps. (2019).
9. L. Moore *et al.*, The mutational landscape of normal human endometrial epithelium. *Nature* **580**, 640-646 (2020).
10. A. L. H. Webster *et al.*, Genomic signature of Fanconi anaemia DNA repair pathway deficiency in cancer. *Nature* **612**, 495-502 (2022).
11. E. Talevich, A. H. Shain, T. Botton, B. C. Bastian, CNVkit: Genome-Wide Copy Number Detection and Visualization from Targeted DNA Sequencing. *PLoS Comput Biol* **12**, e1004873 (2016).
12. M. Lawrence *et al.*, Software for computing and annotating genomic ranges. *PLoS Comput Biol* **9**, e1003118 (2013).
13. G. Marsico *et al.*, Whole genome experimental maps of DNA G-quadruplexes in multiple species. *Nucleic Acids Res* **47**, 3862-3874 (2019).
14. J. Hon, T. Martinek, J. Zendulka, M. Lexa, pqsfinder: an exhaustive and imperfection-tolerant search tool for potential quadruplex-forming sequences in R. *Bioinformatics* **33**, 3373-3379 (2017).
15. I. R. Hahne F, Visualizing Genomic Data Using Gviz and Bioconductor. *Statistical Genomics: Methods and Protocols* http://dx.doi.org/10.1007/978-1-4939-3578-9_16, 335-351 (2016).
16. B. Gel *et al.*, regioneR: an R/Bioconductor package for the association analysis of genomic regions based on permutation tests. *Bioinformatics* **32**, 289-291 (2016).
